# Electrochemical and computational evaluation of hydrazide derivative for mild steel corrosion inhibition and anticancer study

**DOI:** 10.1038/s41598-024-70715-w

**Published:** 2024-09-10

**Authors:** Hany A. Batakoushy, Saeyda A. Abouel-Enein, Reham M. M. Morsi, Hanem M. Awad, Basma Ghazal, Howida S. Mandour

**Affiliations:** 1 Department of Pharmaceutical Analytical Chemistry, Faculty of Pharmacy, Menoufia University, Shebin Elkom, 32511 Egypt; 2 Department of Chemistry, Faculty of Science, Menoufia University, Shibin El Kom, Egypt; 3 Physical Chemistry Department, National Research Centre, 33 El Bohoth St., Dokki, P.O. 12622, Giza, Egypt; 4 Department Tanning Materials and Leather Technology, National Research Centre, Giza, Egypt; 5 Organometallic and Organometalloid Department, National Research Centre, Dokki, Cairo, 12622 Egypt

**Keywords:** Mild steel, Inhibitor (HL), Electrical conductivity, DFT study, Anticancer, Fluorescence, Saline medium, Chemical biology, Chemistry, Physics

## Abstract

In the present study the authors’ main goal is to avoid the corrosive attack of the chloride ions of 3.5% NaCl solution in saline medium on the mild steel (MS), by addition of small amount of a new derivative of the hydrazide called ligand (HL), as a corrosion inhibitor. This study had been achieved by employing different electrochemical measurements such as, open circuit potential (OCP), electrochemical impedance spectroscopy (EIS) and potentio-dynamic polarization (PDP) methods. The results of the electrochemical test (OCP), showed that, the open circuit potential of the mild steel in saline solution, was guided to more positive direction in presence of the ligand (HL), at its ideal concentration (1 × 10^−3^ M), compared to the (OCP), of the mild steel in absence of (HL). The results of the electrochemical methods, EIS and PDP presented that, the ligand (HL), was acted as a good corrosion inhibitor for hindering the corrosion process of the mild steel in 3.5% sodium chloride, as it was recorded a good percentage of the inhibition efficiency (77.45%, 53.41%, by EIS and PDP techniques respectively), at its optimum concentration (1 × 10^−3^ M). Also, the corrosion rate of the mild steel in the saline medium without (HL), was listed about (0.0017 mm/year), while in existence of (HL), was decreased to a value about (0.00061 mm/year). As well, some of electrical properties of (HL), and its derivative [Pd(II), Cr(III), and Ru(III)], complexes were investigated such as; the activation energy (E_a(ac)_), which recorded values in the range of 0.02–0.44 (eV) range and electrical conductivity which listed values at room temperature in the range of 10^−5^–10^−8^ S.cm^−1^. The results of the AC and DC electrical conductivity measurements for (HL), and its derivative [Pd(II), Cr(III) and Ru(III)] complexes indicate semiconducting nature which suggests that these compounds could be used in electronic devices. Also, the complexes exhibited higher conductivity values than (HL). Photophysical studies showed good florescence properties of HL that indicated that it can be used to determine most of the drugs with no fluorescence properties by quenching and calculating quantum yield. Moreover, the hydrazide ligand (HL), has shown selectivity as an active anticancer candidate drug for both breast and colon cancer in humans. Density function theory demonstrated that, the frontier molecular orbital HOMOs of the complexes have exhibited similar behavior and the charge density has localized in the metallic region of all the studied complexes. Also, the values of the energy gap of the ligand (HL), and its complexes Pd(II), Cr(III) and Ru(III), had been arranged in this order HL > Cr(III)** > **Ru(III) > Pd(II). All characterization using different spectroscopic techniques were reported to elucidate the proposed structures such as; thermal analysis, elemental analysis of C, H, and N atoms, spectral analysis using IR, UV, ^1^H NMR techniques, scanning electron microscopy and energy dispersive X-ray analyses.

## Introduction

Since the discovery of metals and their alloys have been used in all aspects of our life’s and industrial applications. Mild steel is considered one of the most commonly metals has been used in the many scopes such as; petroleum, industrial, construction, and building sectors in the form of, a plates, bars, and pipes. But the majority of these metals are thermodynamically unstable in their pure state and are not in isolation or separation from the surrounding environment; therefore, they interact or impacted by those environmental factors like; temperature, pressure, humidity, salinity, and rain beside the negative effects that, resulted from poor human dealings such as, acid washing, acetification and chemical process in industry, all these conditions led to the presence of a big and serious problem; corrosion process^1^.

This harmful corrosion process leads to economic loss by consuming or dissolving of these metals in the surrounding environment. Controlling and treating of these environmental factors are hard but, the metals can be protected by several methods, one of them using of inhibitors^2,3^. These inhibitors, which are known as the substances added in small amounts to retard or prevent the actions of the corrosive environmental factors on the metals by forming a protective layer on their surfaces. These inhibitors include many compounds such as, inorganic, biopolymers, bio-surfactants, vitamins, tannins, and other natural and organic compounds^4^. These compounds must be tested in order to ensure that they are safe and not dangerous to the environment or human health^5,6^.

Many evaluation techniques were developed to evaluate the effects of the analytical processes for these compounds on the environment and on human health^7^. Analytical eco-scale assessment (ESA), the most widely used tool, is considered an eco-friendly analytical approach that, achieves a balance between being a sensitive method on one side and not affecting the environment on the other^8^. Because of their economy, efficiency, ecology and environmental friendliness, organic compounds are regarded as excellent inhibitors of corrosion^9,10^.

Organic compounds containing aromatic rings, heterocyclic rings, double bonds, triple bonds, polar functional substituents, and electron-donating substituents (EDS), which enhance and raise the inhibitory efficacy of these organic compounds to prevent the corrosion process^11–16^.

In previous studies, it was found that, the derivatives of pyrazole, triazole and heterocyclic compounds such as azole derivatives which contains donor heteroatoms like; nitrogen, sulfur and oxygen atoms, which increases the capability of these compounds to be a good corrosion inhibitor^17–19^. Also, hydrazide derivatives are organic compounds which have been greatly used as a good inhibitor to control and impeded the bad effects of the redox reactions of the aggressive media like; sulfuric acid, nitric acid, hydrochloric acid and sodium chloride on the metals such as, mild steel^20^.

Especially sodium chloride solution, which his percentage in sea water was estimated about (3.5%), (its salinity), therefore, it has a bad effect on metals, particularly mild steel, as it is considered a very important metal that is widely used in ship building, water storage tanks, oil tankers, pipelines, and in various industrial applications^21,22^.

The adsorption mechanism of the inhibitor molecules on the interface between the metal and the solution, carried out by four methods: (1) bonding formation between the molecules of the inhibitor and the metal's surface, which is often electrostatic, i.e., physic-sorption; (2) the uncharged electron pairs of the inhibitor molecules interact with the metal; (3) p-electrons interact with the metal by forming a chemical bond, i.e., chemisorption; (4) a mixed type of adsorption, i.e., physio–chemisorption, and the literature indicates that the majority of organic compounds use physio–chemisorption to adsorb^23–25^.

In addition, the importance of the metal complexes cannot be overlooked as they have many uses in various domains such as, analytical chemistry, biological activity, electrochemical and catalytic applications^26,27^. And, as it was studied previously^28^, that the importance of the metal complexes was attributed to their structure and electronic distributions. For instance, synthesized ruthenium and chromium complexes exhibited good redox and emission properties which were used as catalysts in the green synthesis of 2-methyl-1,4-naphthoquinone (vitamin K3) from 2-methylnaphthalene^26^. Also, it was listed before that, synthesis of palladium complex and palladium based nanomaterials played important role in different electrochemical objective such as, capacitors, biosensors, gas sensors, hydrogen storage and fuel cell^27^.

In the current work, the main target is to use the synthesized organic compound 2-(3-amino-4,6-dimethyl-1H- pyrazolo [3,4-b] pyridin-1-yl) acetohydrazide (HL), as new organic corrosion inhibitor for mild steel in saline medium 3.5% NaCl. The structural properties of the synthesized hydrazide ligand (HL), and some of its derived metal complexes [Pd (II), Cr (III), and Ru(III)] were elucidated and proved using several methods; spectroscopic techniques (FT-IR, UV–Vis, 1H-NMR, mass), elemental analysis, magnetic moment and thermal analysis. Corrosion behavior, anticancer and greenness evaluation tool of the hydrazide ligand (HL), were carried out using several techniques. Also, the electrical properties and density functional theory (DFT), of the ligand (HL), and its derivative metal complexes [Pd(II), Cr(III), and Ru(III)] have been studied. Scanning electron microscopy and energy dispersive X-ray analysis were used to characterize the surface of the mild steel.

## Experimental data

### Materials and chemicals

The chemicals were all of analytical grade (BDH, Sigma, or Aldrich) and employed without additional purification as received. Spectral analysis (IR, U.V., and ^1^H NMR techniques), thermal analysis (TG and DTG), and elemental analysis (C, H, and N) were measured. All the chemical and the methods used for preparation and characterization of the ligand (HL), had been reported in Figs. S1, S2, S3.

### Preparation of hydrazide derivative; ligand (HL)

Preparation of ligand (HL), 2-(3-amino-4,6-dimethyl-1H-pyrazolo [3,4 b] pyridin-1yl) aceto-hydrazide was done as the following steps:

- (a) The first step preparation of Ethyl2-(3-amino-4,6-dimethyl-1H-pyrazolo[3,4-b] pyridin-1-yl) acetate by adding drops of ethyl chloroacetate to pyrazolopyridine.
- (b) The reaction mixture has been stirred, collected, washed, and recrystallized.
- (c) The derivative (HL), ligand of the hydrazide was prepared by addition of an excess of hydrazine hydrate to 2-(3-amino-4,6-dimethyl-1H-pyrazolo [3,4-b] pyridin-1-yl) acetohydrazide which was previous prepared.
- (d) The pale-yellow hydrazide crystals (0.91 g, 77%) are obtained from the reaction mixture after it’s been refluxed, cooled, collected, dried, and recrystallized from methanol.
- (e) The analytical data displayed that, the percentage of carbon, hydrogen and nitrogen was % 51.13, %5.88, and % 35.47 respectively, and the calculated chemical formula was written as follows: C10H14N6O. Scheme (1), shows the preparation steps of the ligand (HL), as reported before^29,30^, (1) pyrazolo-pyridine (2) ethyl 2-(3- amino-4,6 dimethyl-1H-pyrazolo [3,4-b] pyridine-1-y1 (3) 2-(3-amino-4,6-dimethyl-1H-pyrazolo [3,4 b] pyridin-1yl) aceto-hydrazid.

### Preparation of the metal complexes derived from the ligand (HL)

**Fig. 1:**
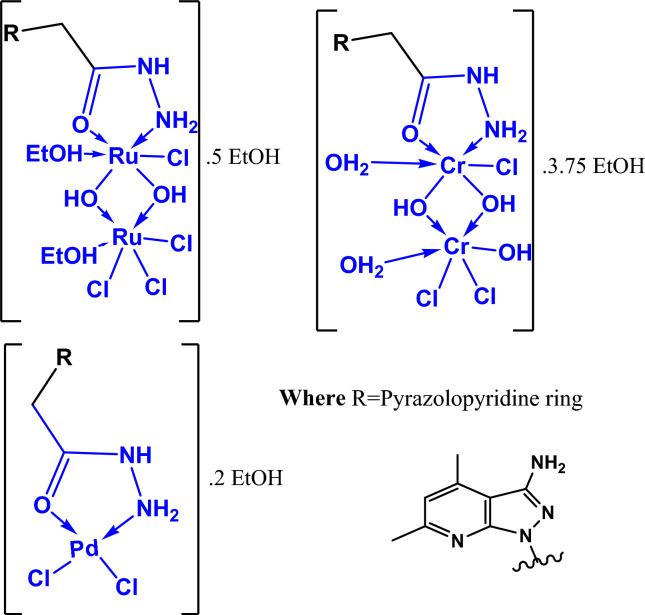
Structure of (Ru (III), Cr (III) and Pd (II)) complexes based on from hydrazide ligand (HL).

The complexes of (M = Pd(II), Cr(III), and Ru(III); n = 0–6) have been obtained through the addition of a heated ethanolic solution of the ligand (5 mL of HL), with an equimolar ethanolic solution (5 mL) of (MCl_2_.nH2O). At 90 ℃, the reaction mixture had been stirred for a few hours before cooling. The produced complex has been filtered out, several times washed with ethanol, and vacuum-dried over anhydrous CaCl_2_. The structure of the metal complexes (Ru (III), Cr (III) and Pd (II), derived from the hydrazide ligand (HL), was shown in Fig. 1.

### Corrosion examination

The corrosion process was achieved onto the mild steel sheet with surface area 1 cm^2^, and its chemical composition was listed in Table 1. A classic three-electrode cell has a working electrode made of mild steel samples, the reference electrode was Ag/AgCl electrode and the counter electrode was platinum wire. An Autolab potentiostat/galvanostat PGSTAT302N was used to perform the electrochemical measurements which includes; the open circuit potential (OCP), potentio-dynamic polarization (PDP) and electrochemical impedance spectroscopy (EIS). The samples of mild steel have been first abraded using emery papers of grades 600, 800, and 1200, followed by acetone to remove any remaining grease, extensively washed with double-distilled water, and lastly dried.

**Table 1:** Chemical composition of mild steel.

Metal	Fe	Mn	Cu	Si	C	P	S
Wt%	98.59	0.40	0.17	0.30	0.11	0.37	0.06

Various concentrations of hydrazide ligand (HL), [1 × 10^−3^, 5 × 10^−5^, 1 × 10^−5^and 5 × 10^−6^ M] were used as a corrosion inhibitor for mild steel in saline solution 3.5% NaCl. For 60 min before each experiment, the mild steel samples were immersed in the selected solution until the steady-state potential (OCP) was reached. After that, the polarization process has been accomplished at room temperature with potential range − 700 to 0.0 mV/(Ag/AgCl), at scanning rate of 1 mV/sec. The working specimen was immersed in the picked solution for 60 min before the electrochemical impedance spectroscopy (EIS) measurements had been done. The measurements were made at a frequency range of 100 kHz–0.01 Hz, and the alternating current (AC) signal was 10 mV peak to peak. The impedance data had finally been resolved and fitted.

### Electrical measurements

Using an LCR Hi-Tester (HIOKI, 3532–50), Japan, at frequencies between 0.042 kHz and 1 MHz and temperatures between 298 and 393 K, the electrical conductivity of the samples under investigation was determined. In close proximity to the specimen, a copper/constantan thermocouple was used for determining the temperature. Using the following expressions, the AC conductivity (ac) is calculated:

$$\sigma_{{{\text{ac}}}} = \omega \varepsilon_{0} \cdot \varepsilon^{\prime } \cdot \tan \delta$$

where ω is the angular frequency, ε_o_ is the free space's permittivity, which is 8.85 × 10^−12^ Fm^−1^, and ε′ is the dielectric constant, which can be calculated using the formula: ε′ = Cd/εo.A, where A is the sample surface area (m^2^) and d is the sample thickness (m). For the complex samples, the capacitance, C, and the dissipation factor, tan δ, are directly acquired from the instrument.

### Fluorescence test

Spectrofluorimetric measurements have been obtained using an FS5 spectrofluorometer (Edinburgh, UK) with a 150 W xenon lamp source for excitement, a quartz cell with a diameter size or surface area of 1 cm^2^, and Fluoracle® software. The scanning speed was set to 1000 nm/min, and the slit widths were 2 nm. A Swiss-made analytical digital balance was employed. A Standard solution (0.1 mg/ml) of the hydrazide ligand (HL), was prepared, then diluted in distilled water to prepare the required final concentrations of the ligand (HL), [5.0 × 10^−7^–4.0 × 10^−6^ µg/ml].

### Anticancer activity

The experimental sections of anticancer activity (materials and methods, cell culture, lactate dehydrogenase (LDH) assay^31^, and statistical analysis**)** were estimated and reported in the experimental section of supporting information.

### Surface morphology

Mild steel samples have been characterized by using a scanning electron microscope (SEM), with energy dispersive X-ray analyses to describe their surface morphology and constituent. These analyses were carried out after the immersion of mild steel samples in a saline solution 3.5% NaCl for about 24 h in presence and absence of the optimum concentration of the hydrazide ligand (HL), 1 × 10^−3^ M.

### Computational details

All computations studies had been carried out using gaussian 16 program^32^ software package. The molecular geometry for the studied compounds was fully optimized using density functional theory B3LYP^33–35^ method and Lanl2dz basis set. No symmetry constrains were applied for optimization procedure. The absence of imaginary frequency had existed from the vibrational analysis at the same level of theory. Frontier molecular orbitals had been studied in the ground state to describe the electronic structural behavior of the synthesized compounds^36,37^. By using HOMO and LUMO energy values for complexes, electronegativity and chemical hardness can be calculated as follows: X = (I + A)/2 (electronegativity), ɳ = (I-A)/2 (chemical hardness), S = 1/2ɳ (chemical softness) where I and A are ionization potential and electron affinity, and I =  − E_HOMO_ and A =  − E_LUMO_, respectively. The optimization process was visualized using Gauss View version 5.0.9. Geometrical parameters (bond lengths and bond angles) of the ligand K and all novel complexes (HL, Ru(III), Cr(III) and Pd(II), had been presented in supporting information Table S2.

## Results and characterization

### Characterization of the ligand (HL) and its metal complexes

The mass and ^1^H NMR spectra of the (HL), had been shown in Figs. S2, S3, and also the physical properties and analytical data of it which represented in previous studies^30^, had been offered in supporting information.

The prepared metal complexes (Pd(II), Cr(III) and Ru(III) exhibited stability and non- hydroscopic properties. These complexes have been found to be readily soluble in dimethyl sulfoxide (DMSO-d6) and N, N-dimethyl-formamide (DMF), and partially soluble in water. All complexes in DMSO (1 × 10^−3^ M) had molar conductance values between (15–36 Ω^−1^cm^2^mol^−1^), which denoting to anions existed in the coordination sphere of the metal ions^38^. The physical properties, elemental analyses and chemical formulae of the metal complexes Pd(II), Cr(III) and Ru(III), had been listed in Table 2, in comparing with the ligand (HL), which confirmed that, the reactions of 2-(3-amino-4,6-dimethyl-1*H*-pyrazolo[3,4-b] pyridin-1-yl) acetohydrazide **(**HL**),** with Cr(III), Ru(III), and Pd(II) chloride salts produced (2 M:1L) and (1 M:1L) molar ratios.

**Table 2:** Physical and analytical data of the hydrazide ligand (HL), and its complexes (Pd(II), Cr(III), and Ru(III)).

Compound	F.W. color	Elemental analysis found/(Calcd.) %				
		C	H	N	Cl	M
(HL).0.25EtOH C_10.5_H_15.5_N_6_O_1.25_	245.782 Pale yellow	51.11 (51.31)	6.02 (6.35)	34.48 (34.19)	–	–
[Pd(HL)Cl_2_].2EtOH C_14_H_26_N_6_O_3_ PdCl_2_	503.804 Yellow	33.88 (33.38)	5.48 (5.20)	16.18 (16.68)	14.51 (14.19)	21.05 (21.11)
[Cr_2_(HL)Cl_3_(OH)_3_(H_2_O)_2_].3.75EtOH C_17.5_H_43.5_N_6_O_9.75_ Cr_2_Cl_3_	704.583 Brown	29.68 (29.83)	6.35 (6.22)	11.89 (11.93)	15.88 (14.12)	14.84 (14.76)
[Ru_2_(HL)Cl_4_(OH)_2_(EtOH)_2_].5EtOH C_24_H_58_N_6_O_10_ Ru_2_Cl_4_	932.754 Black	30.98 (30.90)	6.57 (6.26)	9.63 (9.01)	15.79 (15.22)	22.47 (21.66)

**Fig. 2:**
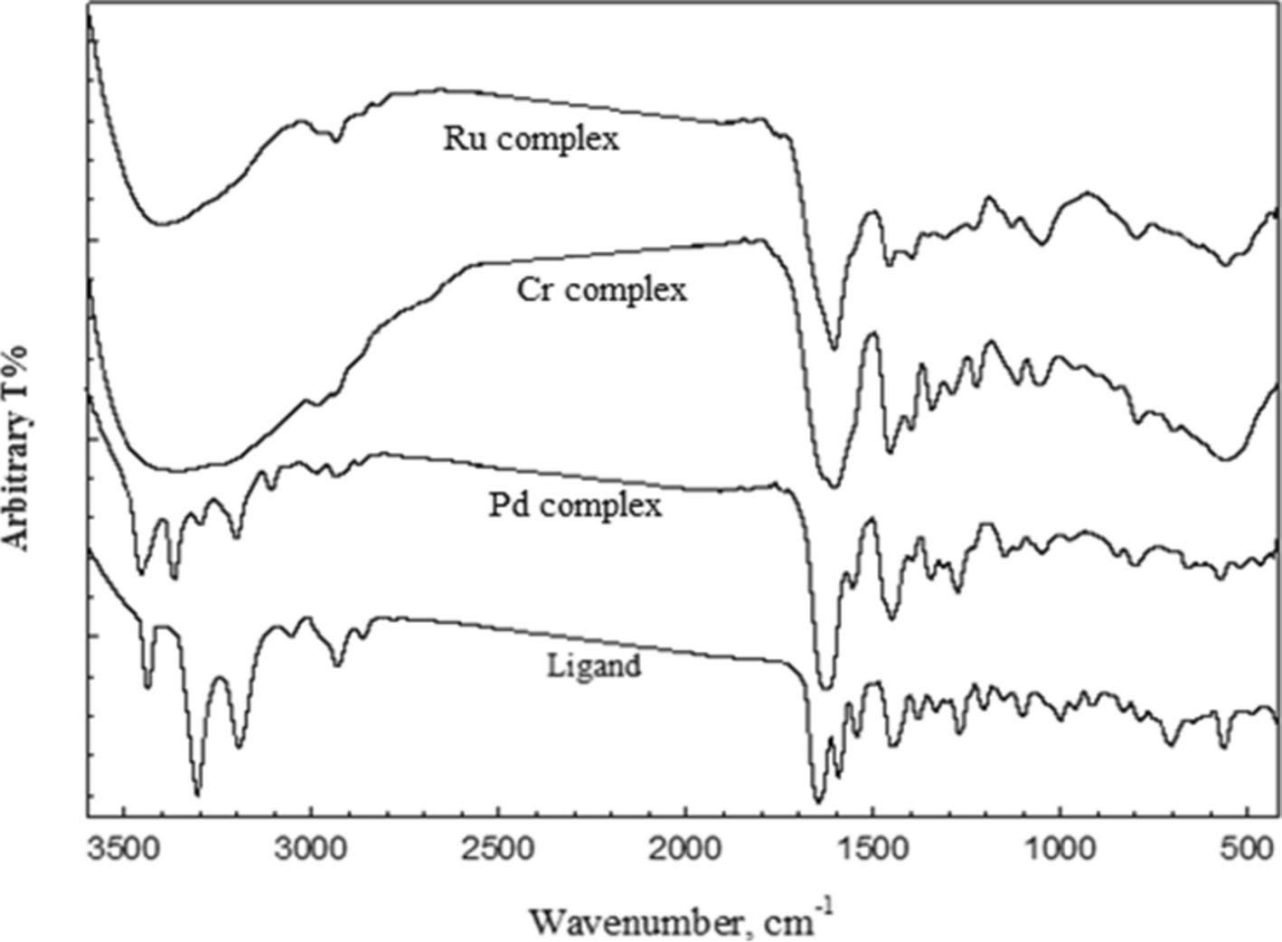
IR spectra of hydrazide ligand (HL), and its (Pd(II), Cr(III) and Ru(III) complexes.

Also, the metal complexes (Pd(II), Cr(III) and Ru(III), had been characterized by using Infrared spectroscopy technique in comparing with the ligand (HL). The obtained data tabulated in Table 3, exhibited stretching bonds in the range 1621–1673 cm^−1^, owing to υ (C = O) bonds asserting with the neutral metal ion keto form. Also, it can be seen that, the bands δ(C = O) and γ(C = O) have changed; they have been shifted in either the negative or positive direction by 6–45 cm^−1^, which is a weak characteristic compared to free ligand. This result suggested that, the oxygen atom existed in the carboxyl group’s in coordination. In addition, the bands appeared in the range of 598–638 cm^−1^, could be attributed to υ (M–O) supporting the above result^39^. It can be noticed from Fig. 2, that, the strong bands υ_*sy*_ (NH_2_) and γ(NH_2_) of the hydrazide ligand at (3306 and 834 cm^−1^), appear in a weak shape and change to a greater or lesser value by 3–56 cm^-1^, after complexation. Additionally, the bands of the amide groups υ (NH) and γ(NH) suffered a shift in the (Pd(II), Cr(III) and Ru(III) complexes by 7–46 cm^−1^, and 66–81 cm^−1^, respectively, compared to the ligand (HL). This result proved that, the hydrazide ligand’s (HL), terminal amino group was engaged in chelation. Moreover, the υ (N–N) underwent complexation from 487 cm^−1^, of the ligand band (HL), to 481–503 cm^−1^ to verify the υ (M–N).

**Table 3:** IR spectral bands and their assignments for (HL), and its complexes.

Compound	υ_as_ (NH_2_)	υ_*sy*_ (NH_2_), γ(NH_2_)	υ_*amide*_ (NH), γ(NH)	υ(C = O)	δ(NH_2_)	υ(N–N)	δ(C = O), γ(C = O) + ring bending	υ(M–O)	ρ(NH_2_), υ(M–N)
(HL).0.25EtOH	3438(m)	3306(s) 834(m)	3194(s) 707(w)	1648(s)	1594 (m)	1002(m) 961(w)	644(w) 563(m)	–	488(w)
[Pd(HL)Cl_2_].2EtOH	3450(m)	3362(m), 823(w)	3194(m) 784(w)	1620(s)	1605(sp.s) 1538(m)	1026(w) 952(w)	686(w) 551(w)	638(w)	503(w)
[Cr_2_(HL)Cl_3_(OH)_3_(H_2_O)_2_].3.75EtOH	3469(w,b)	3362(w,b) 843(w)	3187(w) 778 (w)	1633(sh)	1595(s) 1544(w)	1043(m) 951(w)	684(w) 549(w)	598(sh)	492(sh)
[Ru_2_(HL)Cl_4_(OH)_2_(EtOH)_2_].5EtOH	3408(w,b)	3303(w) 849(sh)	3166(w) 788(w)	1630(sh)	1595(s) 1539(w)	1035(m) 974(sh)	701(w) 545(w)	623(v.w)	496(v.w)

*vw* very weak, *w* weak, *m* medium, *s* strong, *b* broad, *sh* shoulder.

### Studying magnetic moments and electronic spectra

The electronic spectrum of the (HL), and its Pd(II), Cr(III) and Ru(III) complexes was reported in Fig. S4. Table 4, shows the magnetic moments and electronic spectra of the Pd(II), Cr(III), and Ru(III) complexes. The electronic spectra of the diamagnetic Pd(II) complex show three d–d transition bands at 522, 488, and 454 nm, which are attributed to the transitions ^1^A1_g_ → ^1^A2_g_ (υ1), ^1^A1_g_ → ^1^B1_g_ (υ2), and ^1^A1_g_ → ^1^E_g_ (υ3), respectively. These bands indicate a square planar Pd(II) complex^40,41^.

**Table 4:** Electronic spectra and magnetic moment values of (HL), and their complexes (Pd(II), Cr(III) and Ru(III).

Compound	Electronic spectra bands (nm)	Assignment	µ_eff._ (B.M.) (per metal ion)
(HL).0.25EtOH	378, 360, 327,309, 253,	n–π* π–π*	–
[Pd(HL)Cl_2_].2EtOH	522, 488, 454, 359,307, 250,	^1^A_1g_ → ^1^A_2g_ ^1^A_1g_ → ^1^B_1g_ ^1^A_1g_ → ^1^E_g_ Intraligand transition	Diamagnetic
[Cr_2_(HL)Cl_3_(OH)_3_(H_2_O)_2_].3.75EtOH	689, 569, 449, 355,316, 252,	^4^A_2_g → ^4^T_1_g(P) ^4^A_2_g → ^4^T_1_g(F) ^4^A_2_g → ^4^T_2_g(F) Intraligand transition	2.38
[Ru_2_(HL)Cl_4_(OH)_2_(EtOH)_2_].5EtOH	766, 490, 356,306, 258,	^2^T_2_g → ^2^A_2_g LMCT Intraligand transition	1.22

The Cr(III) complex’s electronic spectrum reveals three d-d transition bands at 689, 569, and 449 nm, which are assigned to the ^4^A_2_g → ^4^T_1_g(P), ^4^A_2_g → ^4^T_1_g(F) and ^4^A_2_g → ^4^T_2_g(F), respectively. These transitions indicate an octahedral geometry^42^. The octahedral geometry and the magnetic moment value of the Cr(III) complex (2.38 B.M.) are compatible.

Two bands at 766 and 490 nm, attributable to the ^2^T_2g_ → ^2^A_2g_ transition and the LMCT, respectively, are displayed in the electronic spectra of the Ru(III) complex, indicating an octahedral geometry^43^. Octahedral complex matches the magnetic moment of Ru(III) complex (1.22 B.M.). The complexes register d-d transitions and additional π–π* and n–π* transitions.

### Thermal studies

**Fig. 3:**
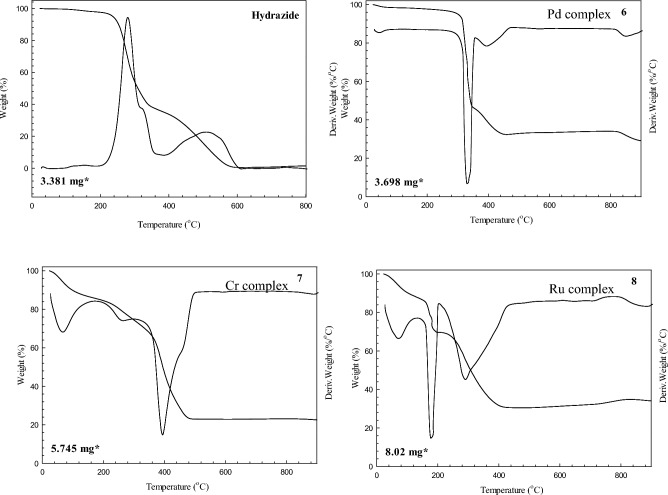
TGA and DTG curves of the ligand (HL), and its metal of the complexes Pd(II), Cr(III) and Ru(III).

The pathways of thermal decomposition step and their assignments for the Pd(II), Cr(III) and Ru(III) complexes are depicted in Fig. 3, and collected in Table 5, after the TG and DTG thermal analyses have been done. The metal complexes Cr(III) and Ru(III) have been decomposed in three steps. The initial thermal decomposition step happened between 26 and 206 °C, and it was controlled by the loss of weight assigned to partial de-solvation. The second step showed weight losses up to 206–320 °C, which corresponded to the elimination of the remaining solvent of crystallization in an additional coordinated solvent (H_2_O or/and EtOH). The formation of metal oxides with residues of carbon resulted from the third step of decomposition, which began at 214–320 °C and was completed in the range of 435–499 °C.

**Table 5:** Thermal decomposition of the (HL), and its metal complexes.

Compound	TG range (^ο^C)	DTG peak (^ο^C)	Mass loss %		Assignment	T_S_ (^ο^C)
			Found	Calcd		
(HL).0.25EtOH	24–150 150–204 204–376 376–618	– – 276^e^ 505^c^	– 2.48 61.51 35.37	– 2.34 62.23 35.43	Stable loss of 0.125 mole of EtOH loss of 0.125 mol of EtOH + C_8_H_9_N_3_ loss of (CH_2_CONHNH_2_ ) + ½ N_2_	
[Pd(HL)Cl_2_].2EtOH	23–307 307–359 359–482 At 483 482–814 814–898 At 898	40^ g^ 329^c^ 390f. – – 856 –	4.62 50.32 12.74 32.17 – 4.82 27.96	4.58 50.26 12.52 32.80 – 4.76 27.88	loss of 0.5 mol of EtOH^b^ loss of 1.5 mol of EtOH^b^, Cl_2_ and C_4_H_9_N_4_ loss of C_2_H_5_N_2_ + 0.5 C PdO + 3.5C^r^ Stable zone loss of 2C PdO + 1.5C^r^	307
[Cr_2_((HL)Cl_3_(OH)_3_(H_2_O)_2_].3.75EtOH	23–206 206–320 320–499 At 499	62^e^ 258f. 385^c^ –	16.85 13.09 47.74 22.69	16.35 13.29 47.08 23.28	loss of 2.5 mole of EtOH^b^ loss of 1.25 mol of EtOH, 2 mol of H_2_O loss of mole H_2_O, 1.5Cl_2_, 0.5H_2_ and ligand pyrolysis Cr_2_O_3_ + C^r^	206
[Ru_2_((HL)Cl_4_(OH)_2_(EtOH)_2_].5EtOH	23–137 137–214 214–435 At 435	67^e^ 176^c^ 286^ h^ –	12.36 17.65 39.47 30.79	12.35 17.29 39.69 30.67	loss of 2.5 mol of EtOH^b^ loss of 3.5 mol of EtOH loss of mole of EtOH , 2Cl_2_ and ligand pyrolysis Ru_2_O_3_ + 3 C^r^	137

The TG curve of the Pd(II) complex displayed a weight loss of 4.62% up to 307 °C, attributed to the elimination of 0.5 mol of EtOH. The decomposition steps of Pd(II) complex include two subdivided steps, at 307–359 °C and 359–482 °C. Firstly, the TG curve showed progressive weight loss about (50.32%) related to decomposition of pyrazolo-pyridine moiety and removal of (C_4_H_9_N_4_), in addition to Cl_2_ and 1.5 mol of EtOH. This procedure was linked to a strong DTG peak at 329 °C. Secondly, the weight loss recorded (12.74%) was linked to a broad DTG peak *T*_max_ = 390 °C, corresponding to the decomposition of the acetyl hydrazide moiety and the elimination of (C_2_H_5_N_2_ + 0.5C). The decomposition ended at 482 °C resulted in formation of (PdO + 3.5C), which led to stable zone within range 482–814 °C. The TG curve showed weight loss at 814–899 °C about (4.82%), due to removal of (2C), leading to formation of (PdO + 1.5C) which confirmed that the percentage of the remainers residue was about (27.96%), and the calculated percentage about (27.88%).

## Corrosion behavior of mild steel in saline solution 3.5% NaCl

### Open circuit potential study

**Fig. 4:**
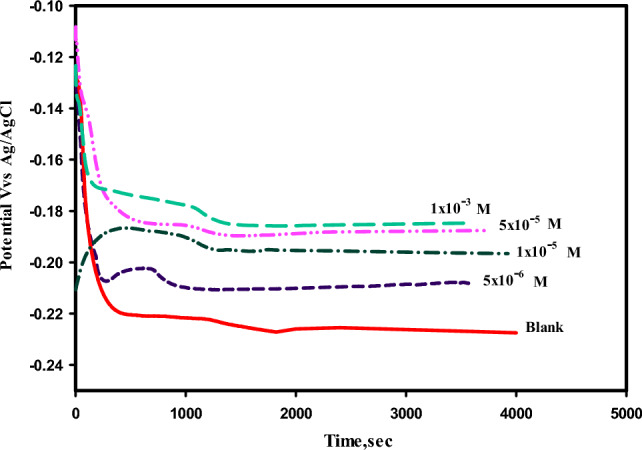
The open circuit potential as a function of time of mild steel in 3.5% sodium chloride solution without and with different concentrations of the inhibitor (HL), recorded at room temperature 25 ºC.

In the present work, the OCP was considered an important method to determine the system’s resting potential, which serves as a function of time after immersion of mild steel samples in 3.5% NaCl solution in absence and presence of the ligand (HL). Figure 4 displayed the difference of the OCP direction from the blank sample MS, free from the ligand (HL), and the samples of MS, with different concentrations of the (HL), [1 × 10^−3^, 5 × 10^−5^, 1 × 10^−5^ and 5 × 10^−6^ M], in saline medium. Obviously, it can be noticed from Fig. (4), that, the OCP was shifted in a positive direction after addition of the ligand (HL), in various concentrations to the 3.5% NaCl solution, recorded the highest value with increasing the concentration of (HL). This result may be attributed to the adsorption of (HL), molecules on the surface of the mild steel, led to blocking the active site of the surface^44^. Therefore, the ligand (HL), can be acted as a good corrosion inhibitor, its type can be related to the value of the displacement of the OCP, which listed a value more than 0.085 mV, so the ligand (HL), can be used as a corrosion inhibitor for both anodic and cathodic reactions^45^.

### Potentio-dynamic polarization

**Fig. 5:**
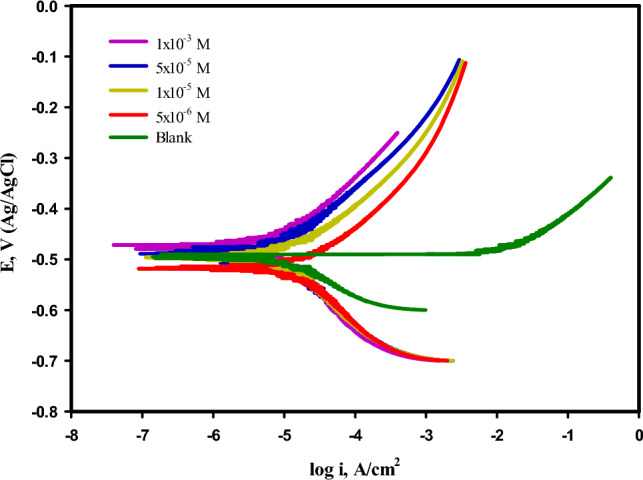
Polarization curves of mild steel in 3.5% NaCl solution without and with different concentration of the (HL), recorded at room temperature 25 ℃.

The PDP method one of the best techniques to deduce the rate of corrosion of the metals and their alloys when subjected to aggressive media, as well as to know the extent of the metal’s efficiency in resisting corrosion process in presence of inhibitors. In the current work, mild steel was exposed to 3.5% NaCl solution, (saline medium), in absence and presence of the inhibitor (HL). The anodic and cathodic polarization curves of mild steel and their calculated parameters are shown in Fig. 5, and Table 6. The effect of adding varying concentrations of hydrazide ligand (HL), as inhibitor to the solution of 3.5% NaCl on the polarization curves of mild steel was clearly shown in Fig. 5, by decreasing the corrosion current density (*icorr*), also it can be noticed from the data listed in Table 6, for example; the (*icorr*), of the uninhibited saline solution recorded value about 17.740 μA cm^−2^, while the concentration of (HL), was added to the same solution; the (*icorr*), was deceased reached the lowest value about 4.001 μA cm^−2^, at the optimum concentration of (HL), 1 × 10^−3^ M. Therefore, it can be said that, the concentration of inhibitor has a big effect on a significant parameters such as; corrosion current density (*icorr*), the percentage of inhibition efficiency $\left(IE\%\right),$ and the corrosion rate, so according to the literature the (HL), can be acted as a corrosion inhibitor for the two reactions hydrogen evolution and metal dissolution i.e. mixed type inhibitor^45^.

**Table 6:** Corrosion parameters of mild steel in a 3.5% NaCl solution without and with different concentration of the (HL), at recorded at room temperature 25 ℃.

	Conc., M	β_a_, mVdec^−1^	− β_c_,mVdec^−1^	− *E*_*co*rr_ mV	${i}_{corr}^{o}$.μA cm^−2^	$IE\%$	C.R., mm/year
Blank	–	114.400	150.900	507.801	17.740	–	0.00171
Various conc. of the inhibitor (HL)	5 × 10^–6^	103.805	140.000	401.602	10.531	40.64	0.00086
	1 × 10^–5^	90.700	120.300	383.350	7.606	57.13	0.00077
	5 × 10^–5^	85.160	107.520	325.210	5.800	67.86	0.00068
	1 × 10^–3^	76.560	97.200	263.032	4.001	77.45	0.00061

In addition, it can be noticed from Fig. 5, that, the Tafel curves were nearly parallel to each other, also the values of β_a_ and β_c_ reported in Table 6, exhibited slight variation than the blank. This result indicates that, the inhibitor worked by blocking the metal’s active regions without impacting the mechanism of the reaction in another meaning, the process of corrosion is simply controlled by hindrance the reaction of the system^46,47^. In general, it was important to use new compounds as inhibitor to protect metals from the bad effects of the corrosion process. In previous studies, authors had been used a novel synthesized compounds with different functional groups or various Substituent to protect mild steel from the corrosive medium such as; imidazothiazole derivatives, imidazopyridines compounds and hydrazide derivatives (N-[(4-methyl-1H-imidazole-5-yl) methylidene]-2-(naphthalen-2-yloxy)^20,48–50^. These compounds gave a good values of inhibition efficiency for protection of MS. The percentage of inhibition efficiency was estimated by using the electrochemical parameter; corrosion current density (*i*_*corr*_), which was reported in Table 6, according to the following equation: -

1 $$IE\%=\frac{{i}_{corr}^{o}-{i}_{corr}}{{i}_{corr}^{o}} x100$$

The symbols ${i}_{corr}^{o}$ and ${i}_{corr}$ pointed to the corrosion current density without and with the inhibitor (HL), respectively. It can be seen from Table 6, the direct proportional between the values of the inhibition efficiency $(IE\%)$ and the concentrations of the inhibitor (HL), which gave a maximum value 77.45% at the ideal concentration of (HL), 1 × 10^−3^ M. Also, the corrosion rate of mild steel in 3.5% NaCl solution was decreased to reach a value 0.00061 mm/year at the optimum concentration of (HL), 1 × 10^−3^ M, while the corrosion rate recorded a value about 0.0017 mm/year for the MS sample in the same solution without (HL). In addition, as it can be seen from Table 6, that, the corrosion potential (*E*_corr_), of inhibited solution shifted in a positive direction by a value more than 0.085 mV, with consideration to the value of (*E*_corr_), of the blank. Based on previous studies, the inhibitor (HL), can act as mixed type inhibitor and this result in consistent with the result of the OCP.

### EIS study

**Fig. 6:**
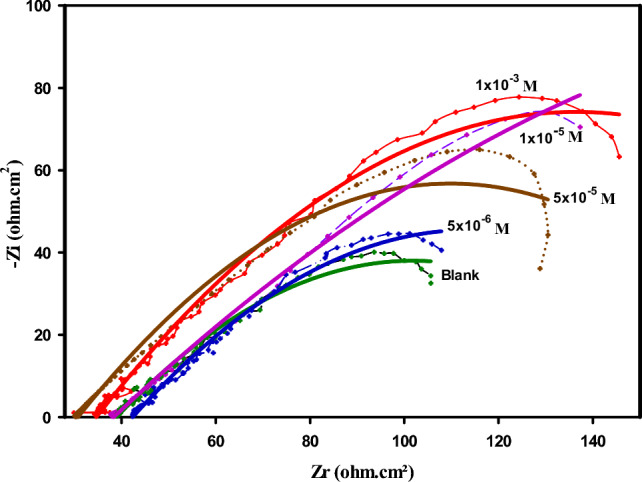
Nyquist plots of mild steel in 3.5% NaCl solution without and with different concentration of the (HL), recorded at room temperature 25 ℃, the symbols refer to the impedance data, the solid line refer to the good fitting.

In order to confirm the results of the potentio-dynamic polarization method, these were done by using another accurate and powerful technique, such as EIS. The influence of the hydrazide ligand (HL), as a corrosion inhibitor in saline medium 3.5% NaCl for the samples of mild steel was deduced and reported in Fig. 6 and Table 7. The Nyquist plots resulted from the impedance data in Fig. 6 showed depressed semicircles plots of the mild steel without and with the inhibitor (HL) in a 3.5% NaCl solution. The deviation of the semicircles of the Nyquist plots for the mild steel than the ideal shape was related to the phenomena called frequency dispersion of the interfacial resistance, also it can be attributed to the roughness, inhomogeneity, impurities, and the random distribution of the active sites on the surface of the working electrode^51^.

**Table 7:** Electrochemical parameters and inhibition efficiency of mild steel in a 3.5% NaCl solution with various concentrations of (HL), recorded at room temperature 25 ℃.

	Conc., M	R_s Ω cm_^2^	R_ct Ω cm_^2^	*CPE*		*C*_dl_ μF cm^2−^	*IE%*
				*Y*_*o* Ω cm_^−2^	*n*		
Blank	–	28.400	270.300	5.01	0.51	11.0 × 10^4^	–
Various conc. of the inhibitor (HL)	5 × 10^−6^	35.810	395.703	1.16	0.56	1.0 × 10^4^	31.69
	1 × 10^−5^	33.410	487.581	0.95	0.59	7.5 × 10^3^	44.56
	5 × 10^−5^	46.800	539.450	0.53	0.61	5.3 × 10^3^	49.89
	1 × 10^−3^	26.020	580.105	0.41	0.64	4.1 × 10^3^	53.41

As well, it can be observed from Fig. 6, that, as the concentration of the inhibitor (HL), increased; the diameter of the semicircle increase, this result confirmed that, the resistance behavior of the metal samples against corrosion process was increased^52^. This behavior was related to the adsorption of inhibitor molecules onto the surface of mild steel; therefore, resulted in formation of a protective layer as previously shown in other study, after formation of a good hydrophobic film (consisting of Ce(OH)3, Ce/Fe-phosphate complexes), St12-steel surface versus the corrosive actions of the chloride ions in seawater^53,54^. Depending on the above result which illustrated the direct proportional between the diameter of the semicircle and the concentration of the inhibitor (HL), beside the parameters deduced from the EIS data and reported in Table 7, these results suggest that, the mechanism of the system (corrosion process), under controlled by the charge transfer resistance (*R*_*ct*_)^55^. Equation (2), was used to calculate the percentage of inhibition efficiency^56^: -

2 $$IE\%=\frac{{{R}_{ct}^{o}-R}_{ct}}{{R}_{ct}^{o}} x100$$

The abbreviation ${R}_{ct}^{o}$ and ${R}_{ct}$ refer to the charge transfer resistance in presence and absence of the inhibitor (HL) respectively.

**Fig. 7:**
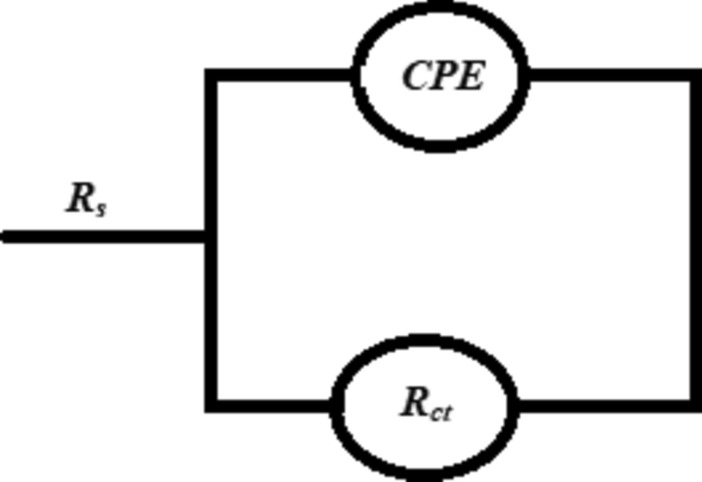
The equivalent circuit employed to fit the impedance data.

The inhibition efficiency recorded the highest value (53.41%), at the ideal concentration of the inhibitor (HL), 1 × 10^−3^ M. The equivalent circuit used to fit the impedance data had been presented in Fig. 7, which contains the solution resistance (*Rs*), the charge transfers resistance (*Rct*), and the constant phase element (*CPE*), which had been used in place of pure double-layer capacitance to more accurate fitting.

The constant phase element (*CPE*) can be defined as follow:

3 $${Q}_{CPE}={Y}_{0}^{-1}{(j\omega )}^{-n}$$

The symbol *Y*_*o*_* is* the constant of *CPE*, j = (-1)^1/2^ which is imaginary number, *ω* is angular frequency and *n* is the *CPE* exponent.

The double-layer capacitance ($${C}_{dl})$$, can be calculated as follow.

4 $$C_{dl} = Y_{0} \left( {\omega \,\max } \right)^{{{\text{n}} - 1}}$$

where ω max is the frequency at maximum impedance and n is the phase shift, its value between (− 1 ≤ *n* ≤ 1), if *n* equal zero, the *CPE* acted as pure resistor, when *n* equal -1, *CPE* represents inductor, and if *n* equal + 1 the *CPE* stands pure capacitor.

The small difference between the real value of the capacitance and its calculated was done by using the following equation:-

5 $$C_{dl} = \frac{1}{{{ }2\pi { }f_{max} R_{ct} }}$$

The abbreviation *f*_max_ expresses the frequency at which the imaginary impedance component is at its maximum.

As, it can be seen from Table 7, that, the double-layer capacitance ($${C}_{dl})$$, had been decreased with increasing the concentration of the inhibitor (HL), which may be related to the displacement of water molecules by the molecules of the inhibitor (HL), resulted in decreasing the double-layer capacitance ($${C}_{dl}),$$ in other words reducing the surface area of the MS which exposed to the saline solution then, decreasing the rate of corrosion^57^. The double-layer capacitance ($${C}_{dl}),$$ can be defined according to the following equation:

6 $${C}_{dl}=\frac{{\varepsilon }^{^\circ }\varepsilon }{d}s$$

where $d$ is the electric double-layer thickness and $s$ is the surface area of the electrode MS.

$\varepsilon$ is the local dielectric constant and ${\varepsilon }^{^\circ }$ is the permittivity of the air.

As per the results of the EIS study, can be seen that it was in agreement with the results of the PDP.

### Surface analyses of mild steel

**Fig. 8:**
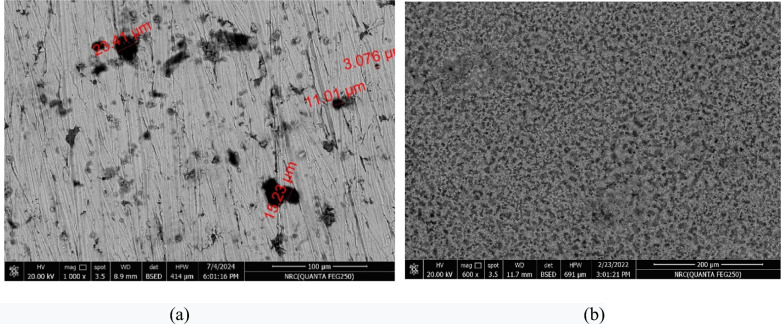
SME images of MS samples after immersion for 24 h in 3.5% NaCl solution (**a**) in absence of the inhibitor (HL) (**b**) in presence of the inhibitor (HL).

The effeteness of the inhibitor (HL), in saline solution 3.5% NaCl as a corrosion inhibitor to protect the sample of MS from the penetration of the chloride ions to its surface causing big damaged had been investigated by using good analytical analyses; scanning electron microscope (SEM) and energy dispersive X-ray (EDX), after immersion the sample of mild steel in absence and presence of the inhibitor (HL), for 24 h. Figure 8a, b, displayed the images of the scanning electron microscope, of the samples MS without and with the inhibitor (HL).

It was cleared from Fig. 8a, that the surface of the metal sample was damaged by the appearance of a large number of pits and cavities with a rough texture. While it became smooth without cracking after the optimum concentration of (HL), 1 × 10^−3^ M was added to saline solution as it was shown in Fig. 8b. This result indicates that, the inhibitor (HL), was acted as a good inhibitor to protect the mild steel sample from dissolution in sodium chloride solution by adsorption of its molecules on the metal surface, resulted in the creation of a protective film on the metal surface, so the corrosion rate was decreased.

**Fig. 9:**
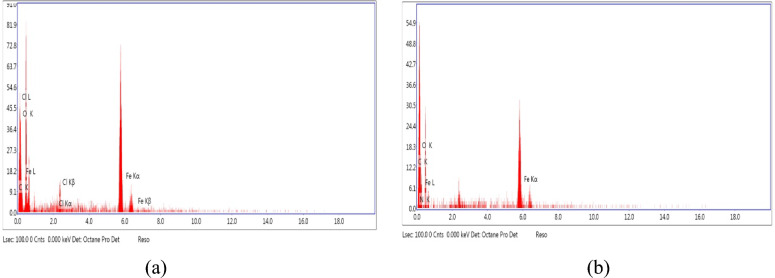
EDX analysis of MS samples after immersion for 24 h in 3.5% NaCl solution (**a**) in absence of the inhibitor (HL) (**b**) in presence of the inhibitor (HL).

This result was confirmed by Fig. 9a, b, and the data of EDX analysis, which reported in Table 8. As, it can be noticed from Fig. 9b that, the appearance of nitrogen peak with weight % 4.14 and atomic % 8.39 was considered an evident for adsorption of the (HL), molecules on the surface of MS. As well, the increasing of the weight % and atomic % of carbon as listed in Table 8, proved the above result.

**Table 8:** The composition elements of the surface of MS after immersion for 24 h in 3.5% NaCl solution in absence and presence of the inhibitor (HL).

Sample	Composition									
	Fe K		O K		C K		N K		Cl K	
	Atomic %	Weight %	Atomic %	Weight %	Atomic %	Weight %	Atomic %	Weight %	Atomic %	Weight %
Blank	75.43	78.53	4.94	9.05	13.99	13.31	–	–	1.51	1.05
MS with 1 × 10^−3^ M of (HL)	23.85	46.83	41.94	23.59	25.49	10.76	8.39	4.14	0.30	0.38

### Electrical studies

**Fig. 10:**
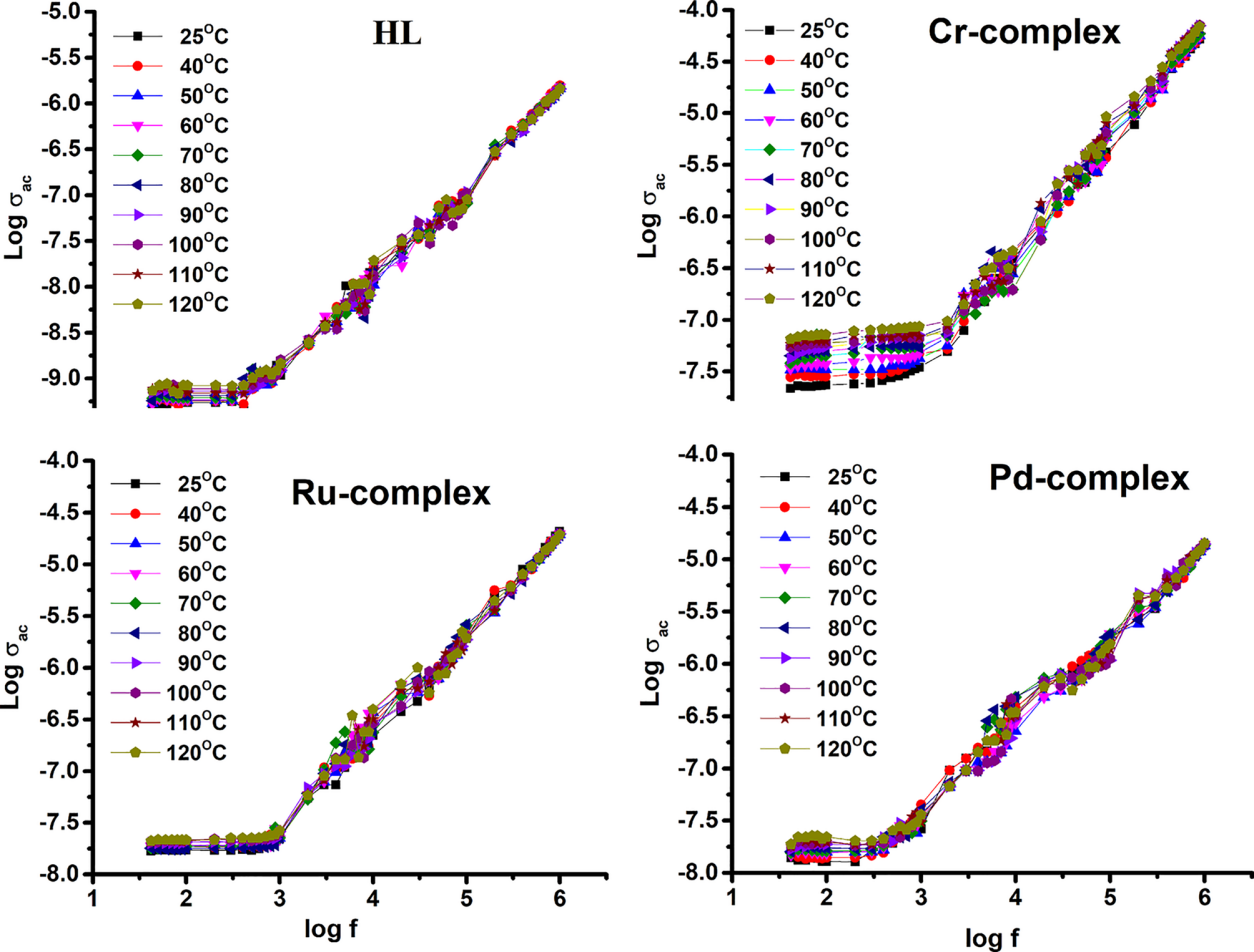
Frequency dependence of the AC electrical conductivity for the ligand (HL), and its metal complexes at various temperatures.

Figure 10 depicts logarithmic plots of conductivity against frequency in the (42 Hz–1 MHz) range at different temperatures. It is clear that conductivity rises as frequency and temperature rise. However, as frequency increases, conductivity becomes less and less temperature dependent. It can be observed that the conductivity for the ligand (HL), Pd(II), complex and Ru(III), complex displays a very slow increasing rate with temperature compared to Cr(III), complex. The conductivity is also seen to be fairly constant at the lower frequency range, which corresponds to DC conductivity (σ_dc_). The σ_dc_ rises as the temperature does in this range of frequencies. This behavior indicates that the electrical conductivity of the material is a thermally activated process.

The conductivity has dispersion at high frequencies, implying that the ions hop in correlated forward and backward motions^58^. In other words, the rise in σ_ac_ with rising frequency indicates that hoping conduction is dominant, and the rise in applied frequency promotes charge carriers’ hopping between the localized states^59,60^. The start of this dispersion changes to greater frequencies as temperature increases, indicating that the onset of ionic hopping contributing to AC conductivity shifts toward high frequency^61^. It is implied that the present samples have semiconducting properties by the gradual increase in AC conductivity with frequency^62^. The electrical conductivity (σ_ac_) values are evaluated at 1 kHz and 1 MHz at room temperature and listed in Table 9, for all samples.

**Table 9:** The DC activation energy (E_a(dc)_), the DC conductivity (σ_dc_) at room temperature (RT) and the AC conductivity (σ_ac_) at 1 kHz, 1 MHz and at RT for the free ligand (HL), and complex samples.

Sample No	E_a(dc)_ (eV)	σ_dc_(S.cm^−1^),RT	σ_ac_(S.cm^−1^),1 kHz	σ_ac_(S.cm^−1^),1 MHz
HL	0.18	5.07 × 10^−10^	1.09 × 10^−9^	1.53 × 10^−6^
Cr(III)-complex	0.52	2.17 × 10^−8^	3.49 × 10^−8^	5.24 × 10^−5^
Ru(III)-complex	0.12	1.69 × 10^−8^	2.74 × 10^−8^	2.10 × 10^−5^
Pd(II)-complex	0.15	1.40 × 10^−8^	2.64 × 10^−8^	1.37 × 10^−5^

**Fig. 11:**
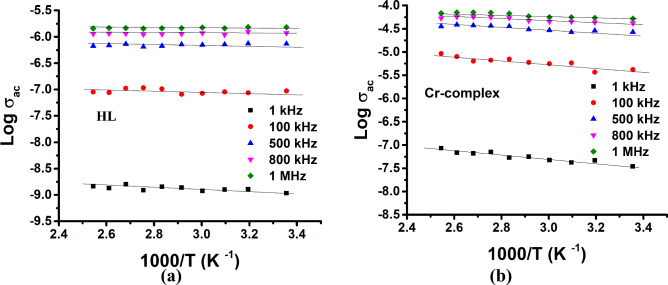
Variation of AC conductivity as a function of the reciprocal temperature for (**a**) the ligand (HL), and (**b**) Cr-complex at different frequencies.

Figure 11 uses the hydrazide ligand (HL), and Cr (III), complex (as a representative sample) to show how temperature affects AC conductivity (σ_ac_) at various frequencies. It is observed that as the temperature rises, σ_ac_ rises as well. There is only one slope observable across the full set of measurements, indicating that there is only one conduction mechanism. The activation energy (E_a(ac)_) values of the investigated samples are estimated and given in Table 10. These values are in the 0.02–0.44 (eV) range. It is also clear from Fig. 11, and the data in Table 10, that the slopes of the curves decrease as frequency rises. The E_a(ac)_ values fall as the applied field frequency increases, which may be attributable to electron jump-enhancement between localized states^63^.

**Table 10:** The AC activation energy (E_a(dc)_) for the ligand (HL) and Cr-complex at different frequencies.

Sample No	E_ac_(eV), 1 kHz	E_ac_(eV), 100 kHz	E_ac_(eV), 500 kHz	E_ac_(eV), 800 kHz	E_ac_(eV), 1 MHz
HL1	0.14	0.13	0.08	0.03	0.02
Cr(III)-complex	0.44	0.42	0.32	0.23	0.13

**Fig. 12:**
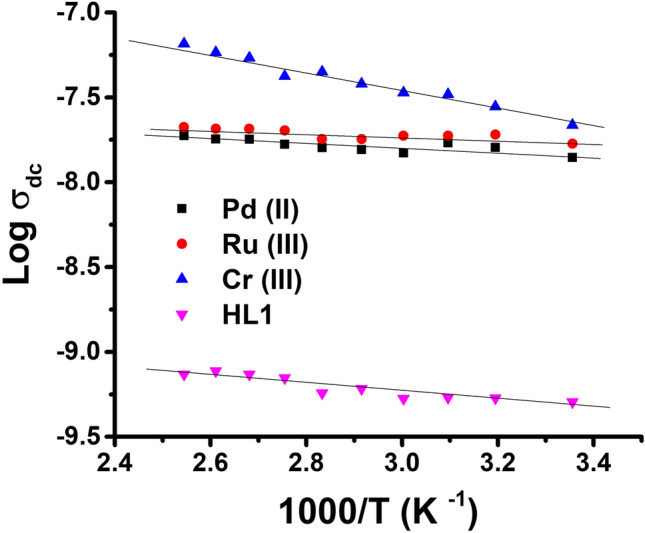
Variation of DC conductivity as a function of the reciprocal temperature for the ligand (HL) and its metal complexes.

By extrapolating the plateau regions detected in Fig. 10, to zero frequency, the samples’ DC conductivity values can be estimated^64^. For hydrazide ligand (HL), and its metal complexes of Pd(II), Cr(III), and Ru(III), the DC electrical conductivity (σ_dc_) of the studied compounds has been plotted against the reciprocal temperature as shown in Fig. 12, It is evident that all compounds have a positive temperature coefficient of electrical conductivity, where the electrical conductivity increases with temperature^65^. The plot of log σ_dc_ against 1000/T reveals a linear relationship with temperature; thus, all samples exhibit semiconducting character across the entire tested range of temperature^66^. All samples are also thermally activated, which may be related to the increased mobility of free charges with temperature^67^. Furthermore, for metal complexes, increased conductivity with increasing temperature can be ascribed to the occurrence of an electronic (d − d*) transition^68^.

As shown in Fig. 12, the plots exhibit Arrhenius behavior that could be described by the relationship: σ_dc_ = σ_0_ exp (E_a(dc)_/kT), where σ_0_ is the pre-exponential factor, k is the Boltzmann constant, E_a(dc)_ is the DC activation energy, and T is the absolute temperature. In Table 9, the estimated activation energies for such samples are listed. It could be observed from Fig. 12, that the sequence of increasing the electrical conductivity of the samples under study at all temperature range increases in the following order: HL ˂ Pd ˂ Ru ˂ Cr.

Metal complexes have higher conductivities than free ligands (HL) due to the presence of transition metal ions in the organic molecules π-electron delocalization during complexation^69^. When organic compounds with transition metals are complexed, the overlap between the metal’s d orbitals and the ligand’s π orbitals results, which extends the delocalization of the π- electronic charges on the hydrazide molecules and facilitates their movement, and subsequently conductivity increases^70^. The investigated metal complexes showed conductivity values at room temperature in the range of 10^−5^–10^−8^ S.cm^−1^; as a result, they may be regarded as semiconducting materials, as it was reported that the electrical conductivity values of semiconductors range between10^−8^ and 10^−3^ S.cm^−1^^67^.

The values of the electrical conductivities (σ_dc_) of the metal complexes may be arranged as mentioned in the above order, depending on the finding that the conductivity increases by increasing the number of unpaired electrons in d orbital of metal ion^68^. The electronic configuration for Cr^3+^: [Ar] 3d^3^ has three unpaired electrons, and for Ru(III): [Kr] 4d^5^ has one unpaired electron, while the metal ion Pd^2+^ has no unpaired electrons as it has a d^8^ electronic configuration, which favors the complex formation with square-planar geometry^71,72^, indicating that its complexes are diamagnetic with 0 unpaired electrons. These numbers of unpaired electrons are confirmed by the electronic spectra and the magnetic assay in Table (3), which is in agreement with the above-mentioned discussion.

Despite that the Cr(III) complex revealed a higher electrical conductivity, its E_a(dc)_ is higher. This higher E_a(dc)_ may be due to that the Cr(III) complex has an extended hydrogen bonding network which includes coordinating water molecules.

## Fluorescence spectroscopy

**Fig. 13:**
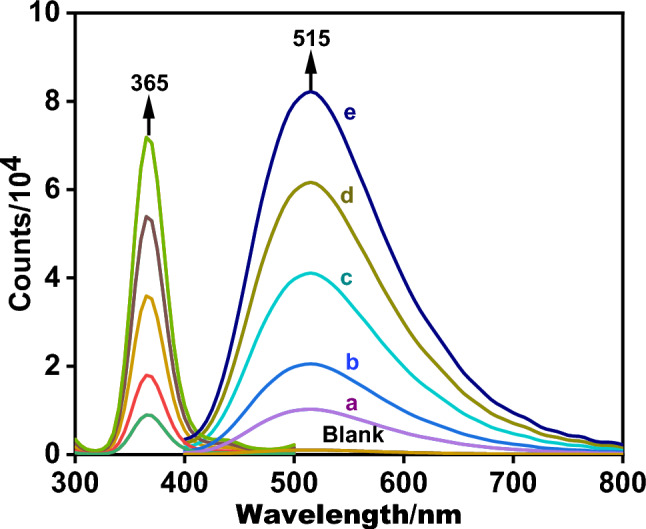
Fluorescence spectra of hydrazide ligand HL in range of (5.0 × 10^−7^4.0 × 10^−6^) at emission 515 nm after excitation at 365 nm.

The hydrazide ligand emission spectra (HL), were recorded in distilled water and are shown in Fig. 13. After exhibiting a 515 nm emission wavelength at a 365 nm excitation wavelength, the hydrazide ligand (HL), was discovered to be red-shifted. Additionally, the red shift in emission wavelength might also be caused by the ligand molecule’s amino group. The increase in fluorescence intensity is also caused by the conjugation of the ligand. In Table 11, a statistical regression analysis and the fluorescence emission spectra of the hydrazide ligand (HL), show that it is fluorescent.

**Table 11:** Sensitivity and regression parameters for quantitative determination of hydrazide ligand by the suggested spectrofluorimetric technique.

Parameter	Ligand (HL)
λex (nm)	365
λem (nm)	515
Linearity range (µg/ml)	5.0 × 10^−7^–4.0 × 10^−6^
Intercept (a)	− 144.5
SD of intercept (Sa)	9.891
Slope (b)	383
SD of slope (Sb)	391
Correlation coefficient (r)	0.9996
Coefficient of determination (r^2^)	0.9993
SD of residual (Sy/x)	12.69
Significance F	7.7 × 10^−11^
LOD (µg/ml)	8.5 × 10^−8^
LOQ (µg/ml)	2.5 × 10^−7^

*SD* standard deviation, *LOD* limit of detection, *LOQ* limit of quantitation.

### Effect of diluting solvent

To determine their impact on the hydrazide ligand's fluorescence intensity, several diluting solvents have been investigated. These solvents included acetonitrile, acetone, water, methanol, ethanol, and ethyl acetate. Water was found to be the best solvent to utilize since it had the maximum fluorescence intensity and reliable findings, as shown in Fig. S5.

### Evaluation of method greenness

When it comes to protecting people and the environment from harmful chemicals and the waste they produce when used in industry, researchers wield considerable power. Chemicals and pharmaceuticals are two examples. Green chemistry must be developed and improved on a regular basis. Recent concerns were employed to evaluate the ecological value of the analytical method, including the eco scale scores^73–75^, and the environmental quality methods index score. Researchers evaluated the recommended methodology’s greenness using the eco-scale. The outcome of the eco-scale evaluation is a number that represents the number of penalty points that were assigned and subtracted from 100. These increased the number of risks that were present throughout the research process. The more “green” the procedure, the greater the score (represented by a high number). The novel strategy included no extraction phase, no heating, and consumed < 0.1 kW/h of energy for one sample. Therefore, the suggested method received an eco-scale score of 89 Table S1, indicating that our strategy was environmentally sustainable.

## Antiproliferative activity

**Fig. 14:**
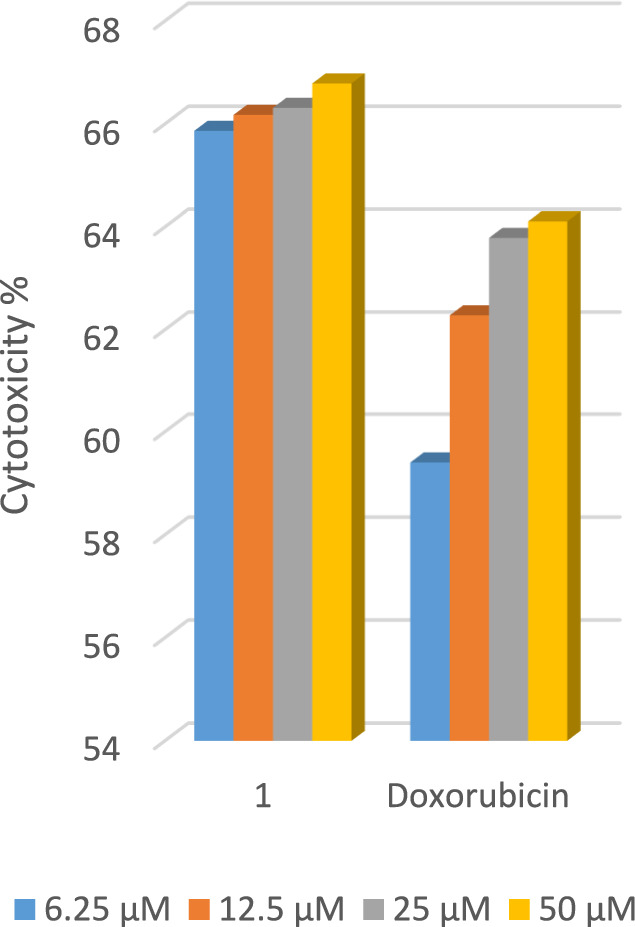
LDH assay results following 48 h of exposure show dose-dependent antiproliferative results of the compounds versus HCT-116 cancer cells.

**Fig. 15:**
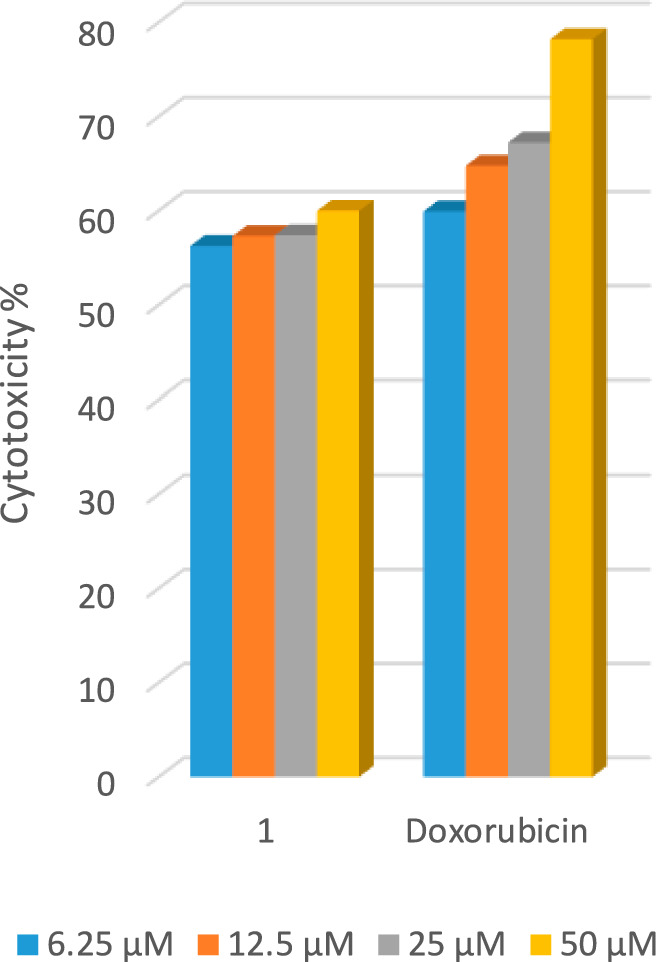
LDH assay results following 48 h of exposure show dose-dependent antiproliferative results of the compounds versus MCF-7 cancer cells.

**Fig. 16:**
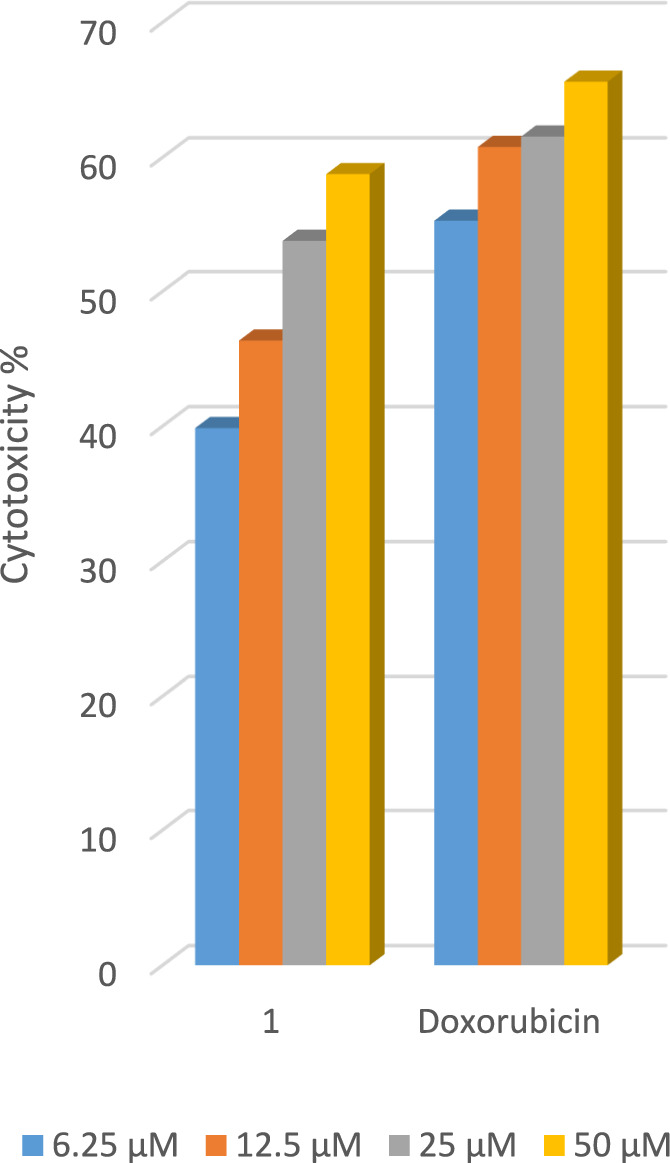
LDH assay results following 48 h of exposure show dose-dependent antiproliferative results of the compounds versus HepG2 cancer cells.

Employing the LDH essay, one component (hydrazide ligand (HL)), was tested in vitro for its efficacy versus the human cancer cells HCT-116, HepG2, and MCF-7. The cytotoxic activity percentages have been computed and compared to the control. Doxorubicin's activity and that of HL versus the three cancer cell lines have been compared. In a dose-dependent manner, the compound inhibited the three types of cancer (HCT-116, HepG2, and MCF-7) Figs. 14, 15, 16. In the instance of human colorectal cancer cell HCT-116, both Fig. 14, and Table 12, show that the hydrazide ligand (HL), has a more potent cytotoxic effect versus HCT-116 than doxorubicin. In the instance of the human breast cancer cell MCF-7, (HL), shows a comparable cytotoxic effect versus MCF-7 when compared to the reference medication in Fig. 15, and Table 12. In the instance of the human liver cancer cell HepG2, HL has a significantly lower cytotoxic effect versus HepG2 than doxorubicin Fig. 16, and Table 12. From the above-mentioned data, one can deduce that the hydrazide ligand (HL), is a selectively active anticancer candidate drug on both the breast and colon cancer types of humans and has less impact on the human liver cancer type.

**Table 12:** The LDH assay’s antiproliferative IC50 for the compounds versus the two lines of cancer cells.

Compound code	IC_50_ (µM) ± SD		
	HCT-116	HepG-2	MCF-7
1-Ligand (HL)	4.7 ± 0.4	7.8 ± 0.5	5.6 ± 0.3
Doxorubicin	5.2 ± 0.3	5.6 ± 0.4	5.2 ± 0.5

## Optimized geometry

**Fig. 17:**
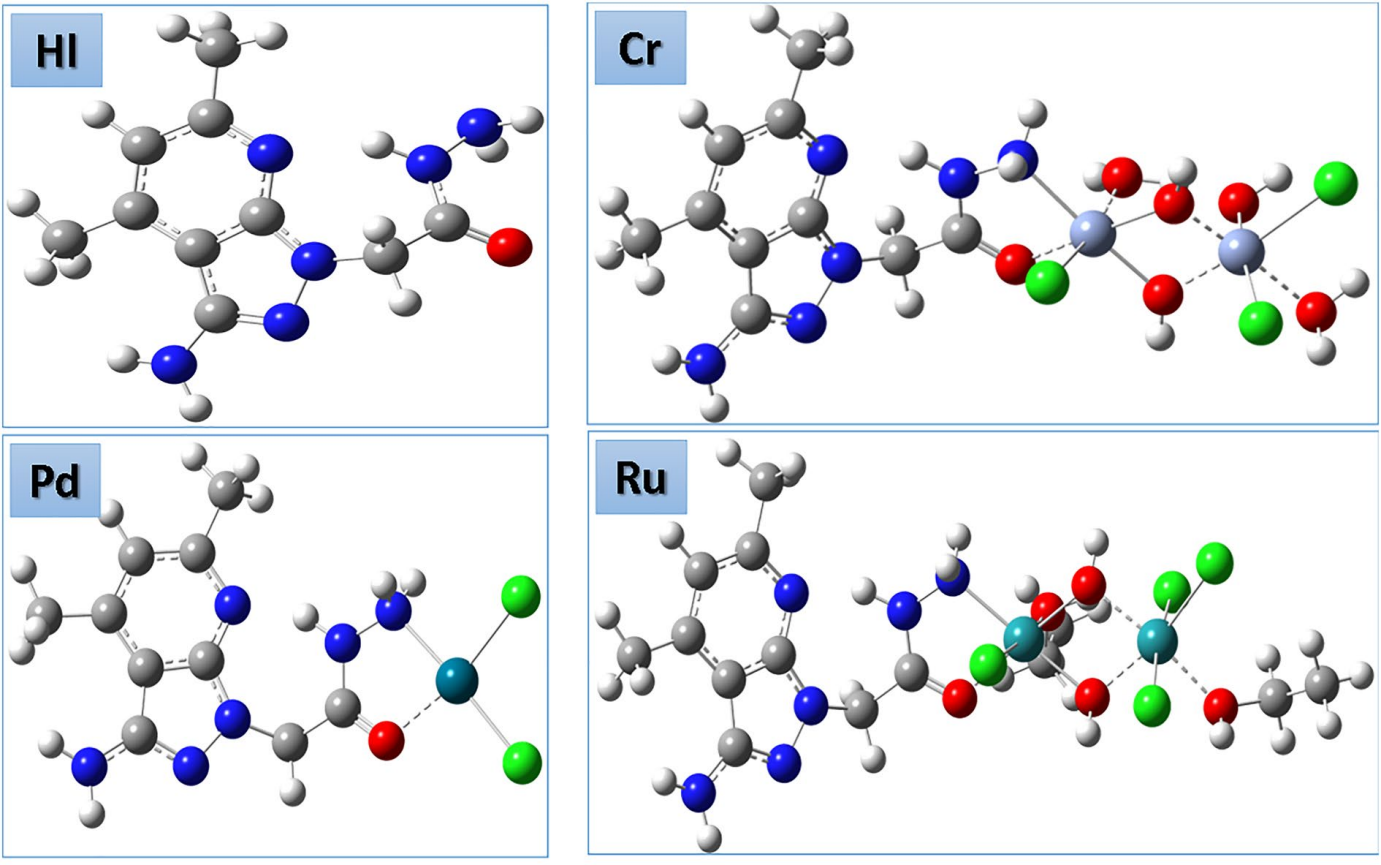
The optimized molecular structure of the ligand K and all synthesized complexes as a ball-and-stick model.

The optimized geometry of the ligand and all complexes were studied in the gas phase via the lanl2dz method. The optimized molecular structures of all compounds are presented in Fig. 17.

### Frontier molecular orbitals and global reactivity descriptor

**Fig. 18:**
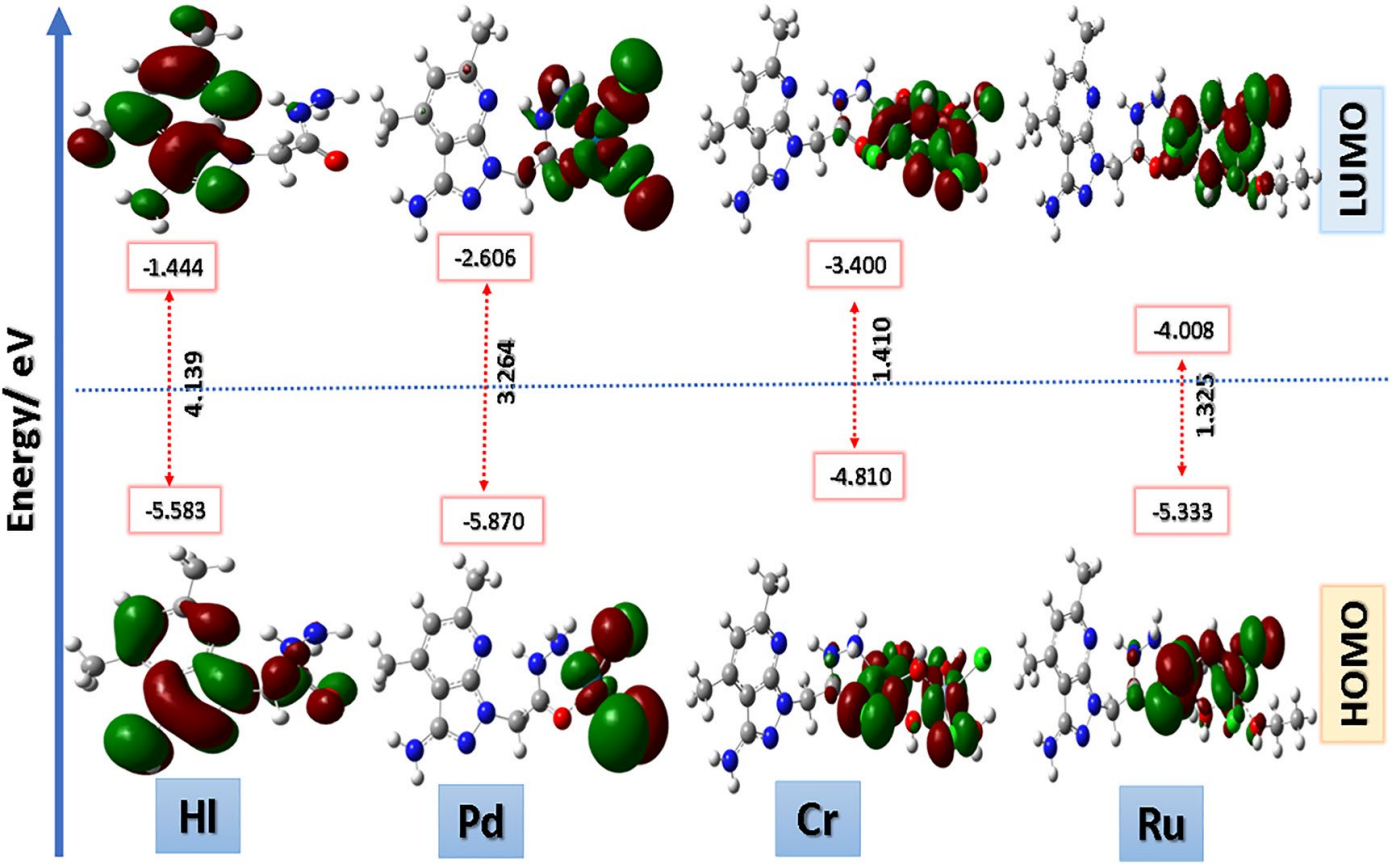
Frontier molecular orbitals for the ligand (HL) and all synthesized complexes calculated in gas phase at the B3LYP/LAN2LDZ method; energy level of HOMOs and LUMOs; and E_gap_ (ΔE).

The structures of all new synthesized fluorinated carbonitrile have been fully optimized via the DFT/B3LYP/6–31G (d,p) level of theory in order to study the electronic behavior of the compounds along with their chemical reactivity. The highest occupied molecular orbital (HOMO) and the lowest unoccupied molecular orbital (LUMO) were studied to show the electronic effects properties based on the DFT studied in order to evaluate the structural properties of the new compounds. The HOMO is related to the electrons in the outermost orbital and tends to donate electrons, whereas the LUMO is the orbital without the outermost electrons and tends to gain electrons. The HOMO energy represents ionization energy, and the LUMO energy represents electron activation energy. The energy difference between the HOMO and LUMO energies is known as energy gab (ΔE), which represents the chemical reactivity and stability of the compounds. The smaller the band gap, the more active the compounds will be. The HOMO and LUMO shapes illustrate the mode of interaction of the synthesized molecules based on electronic interaction properties, which illustrate intramolecular charge transfer. In our work, these parameters will illustrate the reactivity of the compounds after full geometry optimization. The HOMOs and LUMOs were shown in Fig. 18, and their values were presented in Table 13. Herein, we will classify our new materials into three main series based on the active core. In the first category of compounds (**3a–f),** the HOMOs and LUMOs were distributed in different ways based on the electronic design of molecules.

**Table 13:** Theoretical energy calculations and dipole moment of the studied compounds and their interaction products.

ID	Energy (eV)			I	A	η	S	µ	X
	E _HOMO_	E_LUMO_	ΔE						
HL	− 5.583	− 1.444	4.139	5.583	1.444	− 2.070	− 0.483	− 1.222	3.514
Pd	− 5.870	− 2.606	3.264	5.870	2.606	− 1.632	− 0.613	− 1.803	4.238
Ru	− 5.333	− 4.008	1.325	5.333	4.008	− 0.662	− 1.510	− 2.504	4.671
Cr	− 4.810	− 3.400	1.410	4.810	3.400	− 0.705	− 1.418	− 2.200	4.105

In the simplest structures of **3a, 3d,** and** 3e**, the HOMOs were located all over the amino-sulfonated-amino structurally designed molecules, while **3b, 3c,** and **3f.** have good electronically separated HOMOs located at the terminal amino groups. The LUMO for the first group (3a–f) was localized over the tri-fluoro carbonitrile unit with very close energy values and band gap separation (from 3.4 to 3.9 eV). The second group of compounds, **4a-g,** have all electronically separated HOMOs and LUMOs; the HOMOs were located all over the amino-benzyl linker and sulfonated-amino moiety based on their structural design. The LUMOs for all compounds **4a-g** and **7** were localized over the tri-fluoro carbonitrile unit with very closed energy values and band gap separation (from 3.3 to 3.9 eV). Only for compound **7,** the last designed molecule with a small linker (only an NH group), the HOMO was distributed over the whole molecule, resulting in the destabilization of the HOMO energy and a higher band gap value (4.7 eV). Table 13, shows some chemical descriptors that describe the electronic parameters of all new compounds and help to describe the biological activity of the molecules. The values of chemical hardness (η) and softness (σ) are generally computed from the HOMO and LUMO energies. The computed values are tabulated in Table 13.

### Molecular electrostatic potential surface

**Fig. 19:**
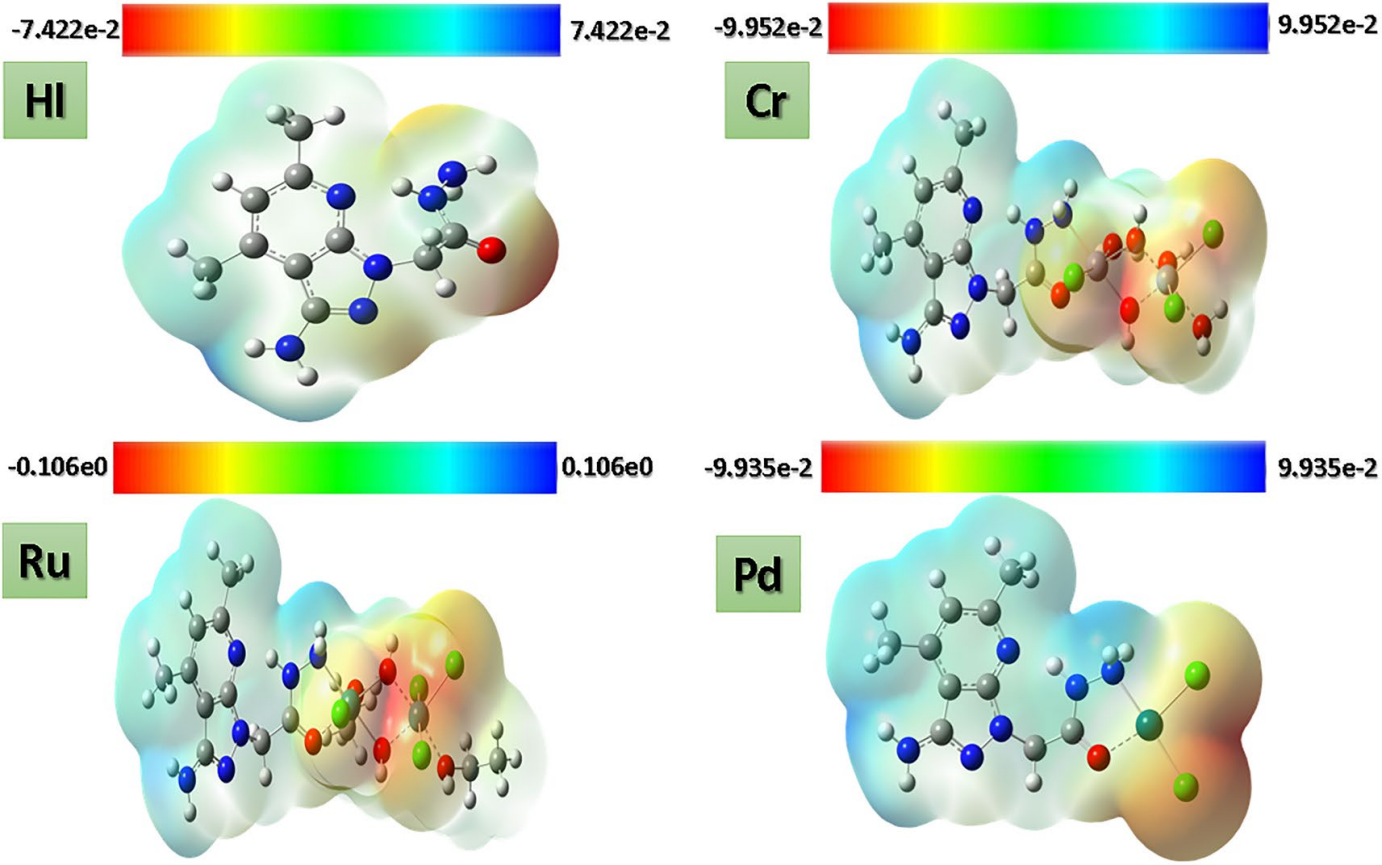
ESP maps for the ligand (HL) and all synthesized complexes by mapping the total density over the electrostatic potential calculated in the gas phase.

Electrophilic and nucleophilic attacks must be known to understand the interaction capability of the newly synthesized compounds. The electrostatic potential surface map had been visualized to show the nucleophilic and electrophilic attacks, as presented in Fig. 19. The negative areas, zero, and positive positions are depicted as red, green, and blue, respectively. The red-colored areas (negative potential) represent the low-energy sites that are usually easy for electrophilic attacks and are located around cyano groups at site 1 and oxygen atoms of sulphonates at site 4. On the other hand, positive potential areas (blue-colored regions) are high-energy regions suitable for nucleophilic attack and mostly appear in the N–H function of site 3 and terminal amine (R’) at site 4, as shown in Fig. 4. The only negative potentials are the sulphonated group and cyano groups; meanwhile, the rest of the compounds are blue in color and, accordingly, have a low electron density around them, hence the very high positive MEP value. From the MEP plots, it was concluded that cyano groups attached to the carbonitrile nucleus and sulphonates influenced the high nucleophilic potential (red), and the other region of the molecule influenced the electrophilic region (blue).

## Conclusions

Through this study, some scientific results can be included as follows:

- 1- The derivative hydrazide ligand (HL), 2-(3-amino-4,6-dimethyl-1H-pyrazolo[3,4-b] pyridin-1-yl) aceto-hydrazide, exhibited good protection for the corrosion process of mild steel in a 3.5% NaCl solution (saline medium), and this was evident through the percentage of inhibition efficiency that was calculated and recorded (77.45%, 53.41%) at the ideal concentration of the inhibitor (HL), 1 × 10^-3^ M and that was proven by the electrochemical methods; PDP and EIS.
- 2- The characterization of Pd(II), Cr(III), and Ru(III) complexes was done by several analyses: analytical analysis (IR, UV, and 1H NMR techniques), elemental analysis (C, H, and N), and thermal analysis, which proved the structures and chemical formulae of these complexes C14H26N6O3 PdCl2, C17.5H43.5N6O9.75 Cr2Cl3, and C24H58N6O10 Ru2Cl4.
- 3- The ligand (HL), and its metal complexes Pd(II), Cr(III), and Ru(III) showed semiconductor behavior based on the DC and AC electrical conductivities also the Pd(II), Cr(III), and Ru(III) complexes exhibited higher electrical conductivity than the hydrazide ligand (HL).
- 4- The suggested spectrofluorimetric approach is simple, precise, and sensitive technique and it is appropriate for use with the prepared hydrazide ligand (HL). It was found that, the ligand (HL), is fluorescent in nature, as seen by the fluorescence emission spectra, so it is very environmentally safe and compliant with green chemistry rules.
- 5- The results of the antiproliferative activity showed that the hydrazide ligand (HL) is a selectively active anticancer therapeutic candidate on both human breast and colon cancer types but less active on human liver cancer types.
- 6- In this work, the lowest unoccupied molecular orbital (LUMO) and the highest occupied molecular orbital (HOMO), structural parameters for the ligand and the studied complexes were calculated via B3LYP/LANL2DZ methods. The frontier molecular orbital HOMOs of the complexes have exhibited similar behavior and the charge density has localized in the metallic region of all complexes. The order of the energy gap values of are in the order Hl > Cr > Ru > Pd. The Cr and Ru complexes have shown the lowest band gab thus the best biological activity.

## Supplementary Information

Supplementary Information.

## Data Availability

All data generated or analyzed during this study are included in this published article [and its Supplementary Information file].
