# Supplementary material for: Electrochemical and computational evaluation of hydrazide derivative for mild steel corrosion inhibition and anticancer study
Source: Sci Rep. 2024 Sep 10;14:21138. doi: 10.1038/s41598-024-70715-w (PMC11387426; doi:10.1038/s41598-024-70715-w)
Supplement: Supplementary file 1 — Supplementary Information. [file 41598_2024_70715_MOESM1_ESM.docx]

**"Electrochemical and Computational Evaluation of Hydrazide Derivative for Mild Steel Corrosion Inhibition and Anticancer Metal Complexes"**

Hany A. Batakoushy^1*^, Saeyda A. Abouel-Enein^2^, Reham M.M. Morsi^3^, Hanem M. Awad^4^, Basma Ghazal^5^, Howida S. Mandour^3^*

*^1^Department of Pharmaceutical Analytical Chemistry, Faculty of Pharmacy, Menoufia University, Shebin Elkom, 32511, Egypt ^2^Department of Chemіstry, Fаculty of Scіence, Menoufia Unіversity, Shibin El Kom, Egypt*

*^3^Physical Chemistry Department, National Research Centre, 33 El Bohoth St., Dokki, P.O. 12622, Giza, Egypt*

^4^Department Tanning Materials &Leather Technology, National Research, Centre, Giza, Egypt.

^5^Organometallic and Organometalloid Department, National Research Centre, Dokki, Cairo, 12622, Egypt.

* Corresponding author [Hany.Batakoushy@phrm.menofia.edu.eg](mailto:Hany.Batakoushy@phrm.menofia.edu.eg)

* Corresponding author [Hmandour77@gmail.com](mailto:Hmandour77@gmail.com)

**Experimental information**

- 1. All chemicals were of analytical grade (BDH, Sigma or Aldrich) and were used as received without further purification. Elemental analysis (C, H, and N) were performed on a Perkin Elmer-2400 elemental analyzer at Main Defence Chemical Laboratory. Also, chloride ions were estimated by using Mohrꞌs method [58]. Infrared spectra were performed on a Nicolet FT-IR spectrophotometer in the range 4000-400 cm^-1^. The ^1^H NMR spectra were recorded in DMSO-*d_6_* on a Varian Gemini 200 NMR spectrometer at 300 MHz. Fast Atom Bombartment (FAB) mass spectra for the ligands were carried out on a Shimadzu Qp-2010 Plus spectrometer. The electron spin resonance (ESR) spectra were recorded on a Varian E-109c spectrometer equipped with a field modulation unit at 100 kHz. The absorption electronic spectra were measured in nujol mulls using a Perkin Elmer Lambda 4B spectrophotometer. Molar conductivity measurements were made in DMSO solutions 10^-3^ M using a type CD6N Tacussel conductimeter. The thermal analysis (TG and DTG) was carried out by using a Shimadzu DAT/TG-50 thermal analyzer with a heating rate of 10^o^C/min under air atmosphere with a flowing rate of 20 mL/min from the room temperature up to 900^o^C using platinum crucibles. Magnetic susceptibilities were measured at room temperature by a modified Gouy method using a Johnson Matthey magnetic susceptibility balance. Diamagnetic corrections were calculated using Pascal̕ s constants. The effective magnetic moments were calculated from the equation μ_eff_ =2.84(*X_M_^corrt^* T)^1/2^. Melting points were measured by using Stuart melting point apparatus.

**Fig. S1.** Preparation of hydrazide ligand (HL)

**Characterization of the ligand (HL)**

1. The physical and analytical data shown that, the prepared hydrazide ligand (HL), was pale yellow in color and the percentage of elemental analyses had been recorded 51.31%, 6.35% and 34.19% for carbon, hydrogen and nitrogen respectively and its molecular weight equal 245.782
2. Mass spectrum of the hydrazide ligand (HL)

The spectrum suggested that the ligand has a monomeric nature. Also, the mass spectrum displayed multiple peaks corresponding to successive degradation of the ligand. The recorded mass spectrum displays that m/z (*I*, %): 234 [M^+^] (14), 174 (100), 161 (24), 147 (14), 133 (13), 105 (11) respectively.


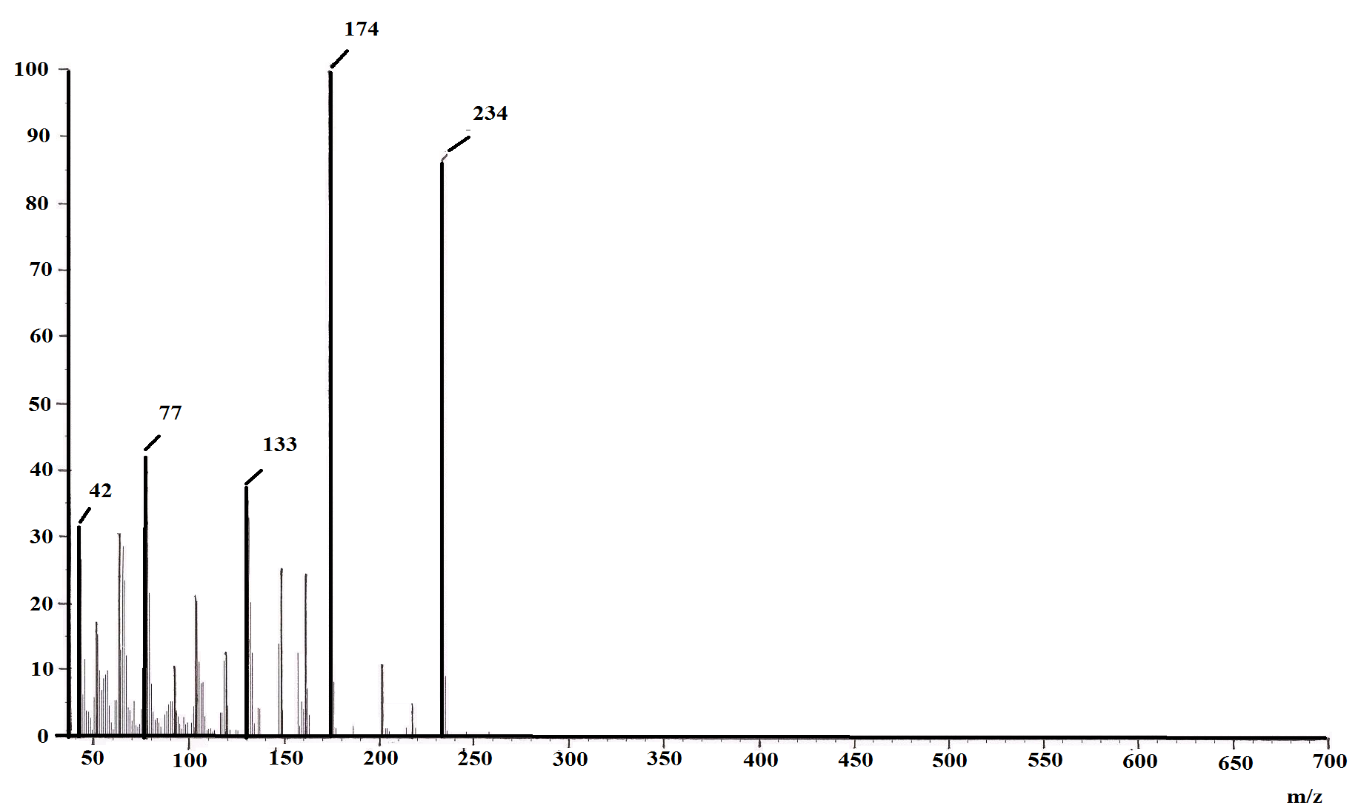


**Fig. S2.** Mass spectrum of hydrazide ligand (HL)

(3) ^1^H NMR spectrum of hydrazide ligand (HL**)**

The spectrum of ligand exhibits signals at *δ*, ppm: 2.43 (3H, s, CH_3_), 2.55 (3H, s, CH_3_), 4.25 (2H, s, NH_2_NH), 4.67 (2H, s, CH_2_), 5.24 (2H, s, NH_2_), 6.63 (1H, s, CH), 9.12 (1H, s, NH_2_NH) respectively.


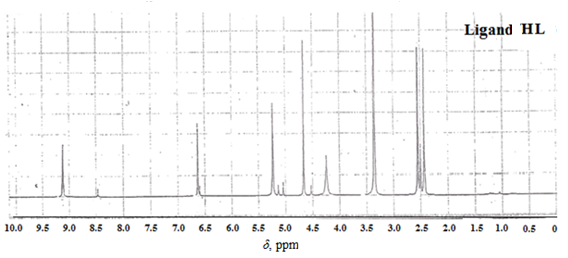


**Fig. S3.** ^1^H NMR spectra of hydrazide ligand (HL**)**

**Fig.S4.** Electronic spectra of hydrazide ligand (HL), and its metal complexes (6) refers to Pd complex (7) refers to Cr complex (8) refers to Ru complex

**Fluorescence test**

**Fig.S5.** Different solvents' effects on the hydrazide ligand's fluorescence intensity; HL at emission 515 nm after excitation at 365 nm

**Table.S1.** calculating penalty points for the developed approach in the greenness evaluation

| Item | Parameter | Penalty Points (Score) |
| --- | --- | --- |
| Technique | Fluorimetry | 1 |
| Reagent | Amount of reagent | 0 |
| Solvent | Distilled water | 0 |
| Temperature | Ambient temperature | 0 |
| Ph | Non | 0 |
| Heating | No Heating | 0 |
| Cooling | No Cooling | 0 |
| Energy | >1.0 | 0 |
| Waste | 1-10 | 6 |
| Occupational Hazards |  | 0 |
| Total Penalty Points (TPPs) |  | 7 |
| ESA score | 100-TPPs | 93 |

**Table S2.** Geometrical parameters (bond lengths and bond angles) of the ligand **K and all novel complexes (Hl, Pd, Ru, and Cr)**

| Hl | 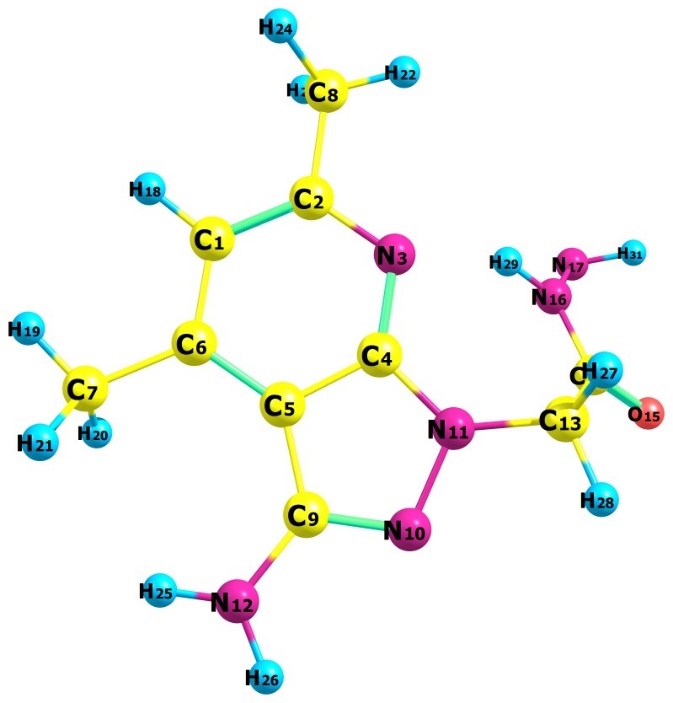 | | | | | | | |
| --- | --- | --- | --- | --- | --- | --- | --- | --- |
| Bond Length | **Exp.** | **Calc.** | **Bond Angles** | **Exp** | **Cal** | **Dihedral Angles** | **Exp** | **Cal** |
| C(4) - N(11) | - | 1.370 | C(4) - N(11) - N(10) | - | 110.762 | N(3) - C(4) - N(11)-N(10) | - | 179.355 |
| C(13) - N(11) | - | 1.462 | N(3) - C(4) - N(11) | - | 126.453 | C(13) - C(14) - N(16)-C(17) | - | 178.335 |
| C(13) - C(14) | - | 1.543 | C(13) - C(14) - N(16) | - | 115.238 |  |  |  |
| C(14) - N(18) | - | 1.434 | C(14) - N(16) - N(17) | - | 121.970 |  |  |  |
| N(16) - N(17) | - | 1.421 |  |  |  |  |  |  |
| Pd (II) | 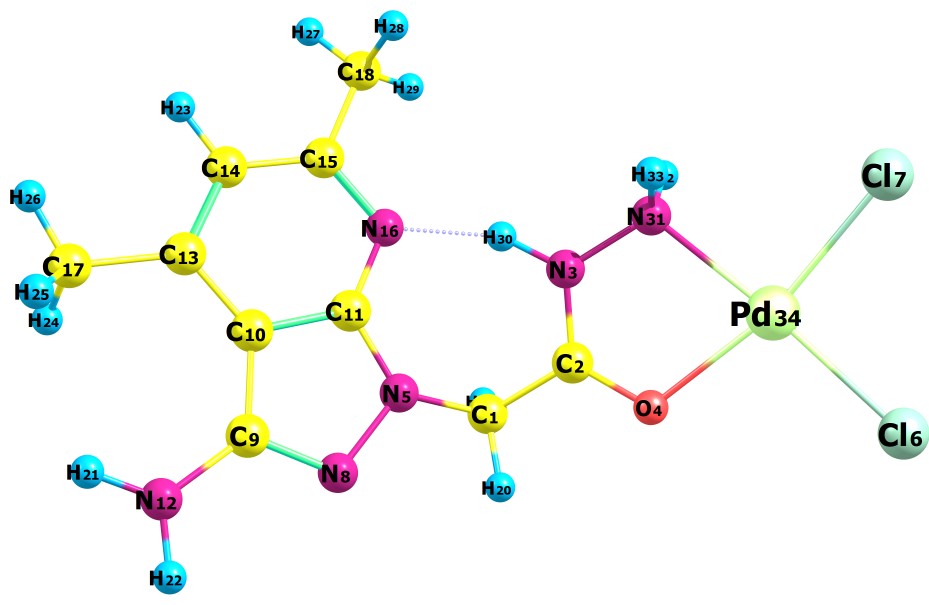 | | | | | | | |
| Bond Length | **Exp.** | **Calc.** | **Bond Angles** | **Exp** | **Cal** | **Dihedral Angles** | **Exp** | **Cal** |
| C(1) - C(2) | - | 1.528 | C(1) - C(2) - N(3) | - | 117.837 | C(1) - C(2) - N(3)-N(31) | - | 178.005 |
| C(2) - N(3) | - | 1.360 | N(3) - C(2) - O(4) | - | 122.749 | N(3) - N(31) - Pd(34)-Cl(7) | - | 177.786 |
| C(2) - O(4) | - | 1.276 | N(31) - Pd(34) - O(4) | - | 78.897 | C(2) - O(4) - Pd(34)-Cl(6) | - | 178.690 |
| N(3) - N(31) | - | 1.440 | Cl(7) - Pd(34) - Cl(6) | - | 95.500 |  |  |  |
| O(4) - Pd(34) | - | 2.149 |  |  |  |  |  |  |
| N(31) - Pd(34) | - | 2.135 |  |  |  |  |  |  |
| Pd(34) - Cl(6) | - | 2.342 |  |  |  |  |  |  |
| Pd(34) - Cl(7) | - | 2.362 |  |  |  |  |  |  |
| Cr (III) | 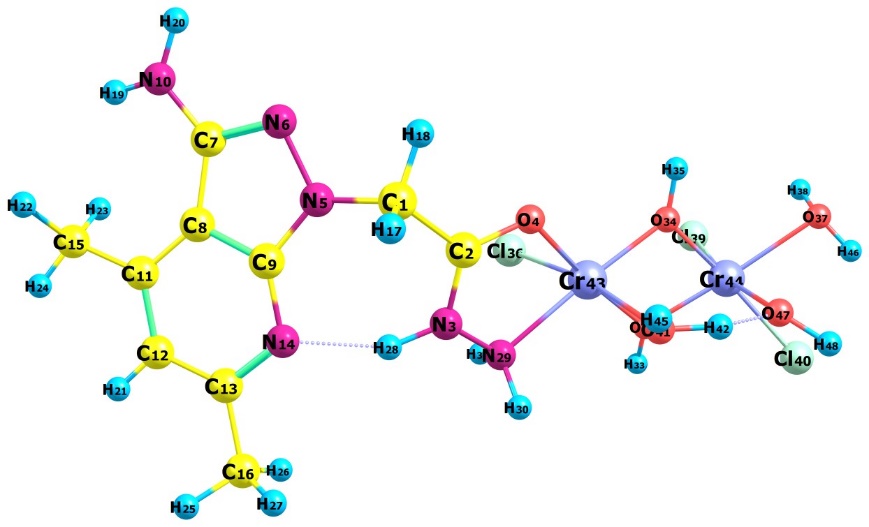 | | | | | | | |
| Ru (III) | 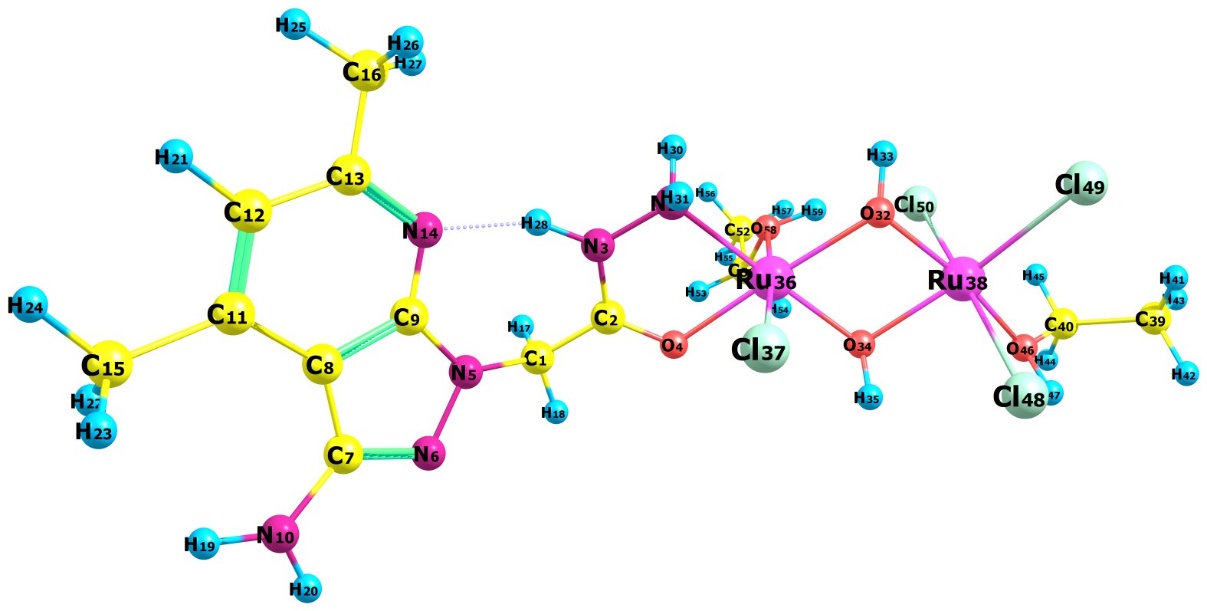 | | | | | | | |

**Anticancer activity**

1. ***Materials and Methods***

Roswell Park Memorial Institute (RPMI) 1640 medium was purchased from Sigma Chem. Co. (St. Louis, MO, USA). Fetal bovine serum (FBS) and fetal calf serum (FCS) were purchased from Gibco, UK. Dimethyl sulfoxide (DMSO) and methanol were of HPLC grade, and all other reagents and chemicals were of analytical reagent grade.

- 1. ***In vitro* anticancer activity:**

**Cell culture**

HepG-2 (Human liver carcinoma), HCT116 (human colorectal carcinoma) and MCF-7 (human breast adenocarcinoma) cell lines were purchased from the American Type Culture Collection (Rockville, MD, USA) and maintained in RPMI-1640 medium which was supplemented with 10% heat-inactivated FBS, 100U/ml penicillin and 100U/ml streptomycin. The cells were grown at 37°C in a humidified atmosphere of 5% CO_2_. All experiments were conducted thrice in triplicate (n = 3). All the values were represented as means ± SD.

**Lactate dehydrogenase (LDH) assay**

To determine the effect of each synthesized compound on membrane permeability in MCF-7, HepG-2 and HCT-116 cancer cell lines, a lactate dehydrogenase (LDH) release assay was used. The cells were seeded in 24-well culture plates at a density of 2 × 10^5^ cells/well in 500 μL volume and allowed to grow for 18h before treatment. After treatment with a series of different concentrations of each compound or Doxorubicin^®^ (positive control), the plates were incubated for 48h. Then, the supernatant (40 μL) was transferred to a new 96 well to determine LDH release and 6% triton X-100 (40 μL) was added to the original plate for determination of total LDH. An aliquot of 0.1 M potassium phosphate buffer (100 μL, pH 7.5) containing 4.6 mM pyruvic acid was mixed to the supernatant using repeated pipetting. Then, 0.1 M potassium phosphate buffer (100 μL, pH 7.5) containing 0.4 mg/mL reduced β-NADH was added to the wells. The kinetic changes were read for 1 min using ELISA microplate reader in absorbance at wavelength 340 nm. This procedure was repeated with 40 μL of the total cell lysate to determine total LDH. The percentage of LDH release was determined by dividing the LDH released into the media by the total LDH following cell lysis in the same well.

- 1. ***Statistical analysis***

All experiments were conducted in triplicate (n = 3). All the values were represented as mean ± SD. Significant differences between the means of parameters as well as IC_50_s were determined by probit analysis using SPSS software program (SPSS Inc., Chicago, IL).
